# A retrospective assessment of forecasting the peak of the SARS-CoV-2 Omicron BA.1 wave in England

**DOI:** 10.1371/journal.pcbi.1012452

**Published:** 2024-09-23

**Authors:** Matt J. Keeling, Louise Dyson

**Affiliations:** 1 The Zeeman Institute for Systems Biology & Infectious Disease Epidemiology Research, School of Life Sciences and Mathematics Institute, University of Warwick, Coventry, CV4 7AL, United Kingdom; 2 Joint UNIversities Pandemic and Epidemiological Research, https://maths.org/juniper/; Stockholms Universitet, SWEDEN

## Abstract

We discuss the invasion of the Omicron BA.1 variant into England as a paradigm for real-time model fitting and projection. Here we use a mixture of simple SIR-type models, analysis of the early data and a more complex age-structure model fit to the outbreak to understand the dynamics. In particular, we highlight that early data shows that the invading Omicron variant had a substantial growth advantage over the resident Delta variant. However, early data does not allow us to reliably infer other key epidemiological parameters—such as generation time and severity—which influence the expected peak hospital numbers. With more complete epidemic data from January 2022 are we able to capture the true scale of the epidemic in terms of both infections and hospital admissions, driven by different infection characteristics of Omicron compared to Delta and a substantial shift in estimated precautionary behaviour during December. This work highlights the challenges of real time forecasting, in a rapidly changing environment with limited information on the variant’s epidemiological characteristics.

## Author summary

One of the key challenges for modellers at the start of any outbreak is to predict its likely scale and the associated burden on the health systems. This was the scenario in December of 2021, when the UK was faced with the invasion of the Omicron (BA.1) variant. For the Omicron BA.1 wave the peak in hospital admission was initially overestimated due to the simplifying assumption that Omicron would behave like a faster spreading version of the Delta variant, and that there would be little or no change to population behaviour during the wave. In this paper, we use a range of models and analyses to understand the challenges of correctly predicting the peak. Compared with forecasts made from the earliest data, the peak was reduced by the shorter infectious period of Omicron, its lower severity but mostly by changes in behaviour and testing over the Christmas holiday season. Historic projections made in December of 2021 that assumed a set of imposed mitigation measures were therefore more accurate than those that assumed no controls, although in practice the controls were largely self-imposed. This paper therefore highlights the challenges of forecasting the likely scale of outbreaks and the importance of understanding the population response to public health messaging.

## Introduction

Real-time modelling of any infectious disease outbreak is a key public health tool, allowing refined planning for future health burdens and an assessment of the likely impact of different controls [1–4]. This real-time modelling process relies on a reliable and regular flow of information, a robust inference scheme to translate the data into estimates of model parameters and then a forward projection mechanism to generate the dynamics while fully accounting for parameter uncertainty. This pipeline from data to outputs should operate in parallel with model development, refining the model structure and assumptions to match the latest epidemiological understanding. Such modelling is probably of greatest use early in an outbreak, when there is great uncertainty about the expected behaviour; however this is also the point when there is greatest uncertainty in the parameter inference, when the model structure and inference schemes are typically not well-defined and often when the flow of data is more erratic. The Omicron BA.1 outbreak in the UK, which occurred during December 2021 and January 2022, therefore offers an idealised test-case for real-time modelling as the model structure, inference mechanisms and data pipelines were all in place.

The Omicron variant of SARS-CoV-2 (B.1.1.529) was first identified in South Africa on 19th November [5] and declared a Variant of Concern (VOC) by the World Health Organisation (WHO) on 26th November. The first cases in the UK were identified in late November, although recent genomic analysis suggests undetected introduction into the UK between 5th and 18th November [6]; the experience in South Africa suggested that there would be a sharp rise in cases leading to another wave of hospital admissions and deaths.

In the UK, analysis of data and modelling projections were presented at three meetings of the Scientific Advisory Group for Emergencies (SAGE meeting 99 on 16th December 2021, SAGE meeting 101 on 23rd December 2021 and SAGE meeting 102 on 7th January 2022), where a scientific consensus was reached on the likely risk posed by the Omicron variant [7–9].

Analysis of the UK data and model-based projections of Omicron from two groups were initially shared with Scientific Pandemic Influenza Group on Modelling, Operational sub-group (SPI-MO) on 1st December and updated on 7th [10]. These early results explored a range of parameters including the transmission advantage of Omicron over Delta and the degree of vaccine escape and suggested large peaks in the number of hospital admissions were possible in the worst case scenarios with limited controls. The model from the London School of Hygiene and Tropical Medicine (LSHTM) peaked at around 7500 admissions per day, while the Warwick model peaked in excess of 10,000—while these worst-case values are extremely large, we will see that they are in keeping with an infection that has Delta-like characteristics but the transmission advantage associated with Omicron. At the time, sensitivity was performed for both stringent controls or reduced severity, both of which were found to limit the peak of the hospital admission wave [10].

In terms of feeding into SAGE and hence into policy, detailed modelling of the Omicron wave by LSHTM produced on 11th December was presented at the 99th meeting of SAGE [7]. This work fit to the early growth of Omicron, and explored a range of assumptions about immune escape and the impact of booster vaccinations; this first modelling work predicted total hospital admissions of 175,000 (CI 139,000-198,000) over the entire Omicron wave (1st December 2021 to 30th April 2022) without additional control measures in the most optimistic case (highly efficacious boosters and limited immune escape). Even with very stringent control measures, the equivalent of the January 2021 lockdown, this early modelling still projected a total of 70,100 (CI 54,000-90,900) hospital admissions during the wave. Peak hospital admissions using the LSHTM model were in the range 5000-10,000 for the most pessimistic assumptions without control, 1500-3700 for the most optimistic case without control and 400-2600 when stringent controls are applied to the optimistic case.

Preliminary results from the Warwick model, again presented at SAGE 99 [7], also concluded that a large outbreak was likely. Peak hospital admissions depended on assumptions about severity and control measures: from 1,500 (CI 590-3,700) admissions per day for strict non-pharmaceutical intervention (NPI) measures and assuming Omicron was only 20% severity compared to Delta; up to 25,000 (CI 17,000-43,000) admissions per day assuming limited controls and the same severity as Delta.

More refined analysis and projections from the JUNIPER consortium, using the Warwick model, were presented to SAGE meeting 101 on 23rd December [8, 11] and was based on case data up to 14th December (due to reporting delays) and hospital admission up to 16th December. At this time Omicron had not yet sufficiently established to lead to a rise in hospital admissions or deaths. The default model assumed that the severity of Omicron compared to Delta was 50%, and considered scenarios with different levels of control—conceptualised as a short-term ‘circuit breaker’ lockdown. The strength of this circuit breaker was set to three different values (by increasing the strength of the precautionary behaviour over a set period); these changes in precautionary between would generate *R*_0_ values of 26% (CI 23-29%), 38% (CI 35-42%) and 68% (CI 66-71%) relative to the pre-COVID mixing. With the weakest controls considered, thought to be comparable with the government’s Plan B, 50% severity was projected to generate a peak of 13,600 (CI 9,300-21,300) daily hospital admissions. The strongest levels of control considered were predicted to generate a January peak of around 1850 (CI 1200-3200) daily hospital admissions. However, it should be noted that without the invasion of Omicron BA.2, which has an even lower estimated severity than BA.1, these early projections show a substantial second wave whenever control measures were relaxed [11].

**Fig 1:**
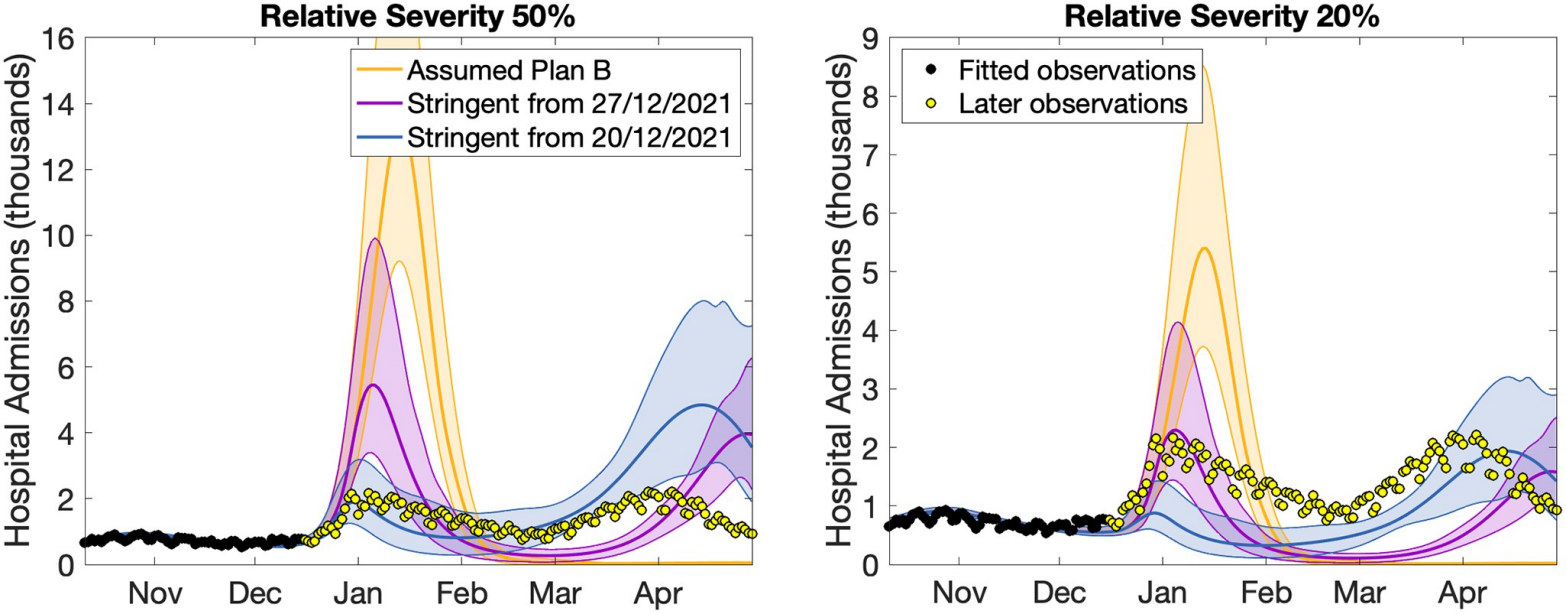
Projections of hospital admissions in England (means and 95% prediction intervals) from the December preprint [11] together with reported admissions. Panel on the left corresponds to a relative severity of 50% (compared to Delta), the panel on the right corresponds to a relative severity of 20%. The three lines correspond to the estimated impact of Plan B (orange), and more stringent controls from either the 27th December (Purple) or 20th December (Blue). The dots represent the data on hospital admissions, both used for fitting (black) and after the fitting period (yellow).

Here we present Figure 8 and 9 from the December preprint [11], and compare the results to observations (Fig 1). As in the rest of this paper, we focus on hospital admissions as our main epidemiological measure. We add the observed number of hospital admissions in England to these December 2021 projections, showing data points used for fitting (black) and later data (yellow). Both sets of projections have Delta-like latent and infectious periods, but differ in the assumed severity (either 50% or 20% of the infection hospitalisation ratio compared to Delta). For both severity assumptions there are scenarios that capture the observed peak, but also scenarios that greatly over-estimate the peak. The challenge was therefore not in generating a model that could approximate the future hospital data, but in choosing between a multitude of different projections any one of which could be plausible; for the Omicron BA.1 wave, this largely corresponded to making the correct assumption about future behaviour.

At the same SAGE meeting [8], LSHTM refined their modelling, showing that imposing moderate control measures (the equivalent of Step 2 in the relaxation roadmap, in place from 12th April to 16th May 2021) would only have a marginal impact on the projected scale of hospital admissions, reducing it by around 10% [12]. Revised peak hospital admissions were in the range 4000-7000 for the optimistic scenario (with or without controls) but increased to 9000-18,000 for the most pessimistic scenarios.

The SAGE 101 meeting [8] also saw two presentations estimating the relative severity of Omicron: a key analysis of data from South Africa (later published as [13]) reporting that the risk of hospital admission for Omicron was only 20% (CI 10-30%) that of the Delta variant; and Report 50 from Imperial College [14] which analysed UK data and estimated a relative severity of 65-89% depending on the partitioning of case and hospital data.

Later JUNIPER consortium and Warwick model projections made at the start of January 2022 [15], although still relying on model fits from 17th December, considered both reduced severity and a shorter generation time—including 50% severity and 33% shorter infection periods. However, these later projections did not include any change to the precautionary behaviour as there had not been any observed change in policy at that point.

Here we combine four different approaches to understand the complexities of predicting the scale of a novel epidemic wave, using Omicron in the UK as a case study. We begin with the theoretical analysis of the simple SIR model, considering how the peak prevalence and incidence are related to key observables: the growth rate of the pathogen, the infectious period and the proportion of the population that are susceptible. This provides a suitable reference to understand the sensitivity of the peak incidence to the fundamental model parameters. We complement this with matching to synthetic data from an SIR model, to assess the ability to infer the key parameters and hence predict the peak level of infection. We then switch our focus to the Omicron wave, and initially consider the proportion of infection due to Omicron during December 2021, fitting the invasion with simple logistic growth models; illustrating the ease with which the growth advantage of Omicron can be captured from early data. Finally, we utilise the type of more complex age-structured model formulated during the Omicron wave, and consider our ability to forecast the peak daily hospital admissions as more data becomes available. For each of these four approaches we give the methods and results together within each section, building a picture of the analysis that can be performed with more bespoke methods and richer data.

## Results

### Simple analysis

For a new outbreak, either due to a novel pathogen invasion or the emergence of a new strain or variant, there are multiple unknown quantities that impact the predicted scale of the epidemic wave. Many of these are difficult, if not impossible, to infer from the early data, especially if the data comes from population-level observations. Detailed data on transmission within households or other closed settings can provide more insights [16–18] but this form of data take longer to collect and the findings are more complex to analyse. The early growth rate, *r*, is easily estimated from population-level observations, with *r* > 0 corresponding to a growing outbreak and higher values of *r* corresponding to a more rapidly growing epidemic with the associated public health consequences. Often this early growth rate is used (together with a measure of the generation time) to compute the basic reproductive ratio (*R*_0_) [19], but the estimation of the generation time is itself complex and generally requires individual level data—often from infection within households.

Here we use a simple SIR-type model to inform how the peak outbreak size scales with the key epidemiological parameters. We take as our starting point, the SIR model ignoring births and deaths, and work with proportions of the population size:

$$\begin{array}{ccc} \frac{dS}{dt} & = & -\beta SI,\\ \frac{dI}{dt} & = & \beta SI\;-\;\gamma I,\\ \frac{dR}{dt} & = & \gamma I. \end{array}$$ (1)

By using proportions (rather than numbers) we remove the need to know the total population size. We further assume that the number of individuals requiring medical attention is proportional to the incidence of infection (*βSI*) with proportionality constant *μ*—although the timing of this medical need will be delayed compared to the timing of infection.

For the SIR model, the early growth rate is given by *r* = *βS*_0_ − *γ* = *γ*(*R*_0_*S*_0_ − 1), where *R*_0_ is the basic reproduction number (*β*/*γ*) and *S*_0_ is the initial proportion of the population that are susceptible to infection. To simplify our notation, we define *ρ* = 1/*γ* to be the average infectious period. We start this model with *R*(0) = 0 so that *R* measures the total proportion of recovered individuals at any point this outbreak; we note that this assumption breaks the usual assumption that *S* + *I* + *R* = 1, instead we have that *S* + *I* + *R* = *S*_0_. This means that the simple SIR model is governed by three parameters: *r*, *ρ* and *S*_0_; with an additional parameter *μ* if we wish to capture the proportion of population requiring medical attention. In the analysis that follows, we assume that the value of *r* has been robustly estimated from early data, but other parameters (*S*_0_, *γ* and *μ*) are uncertain and hence we consider a range of values.

**Fig 2:**
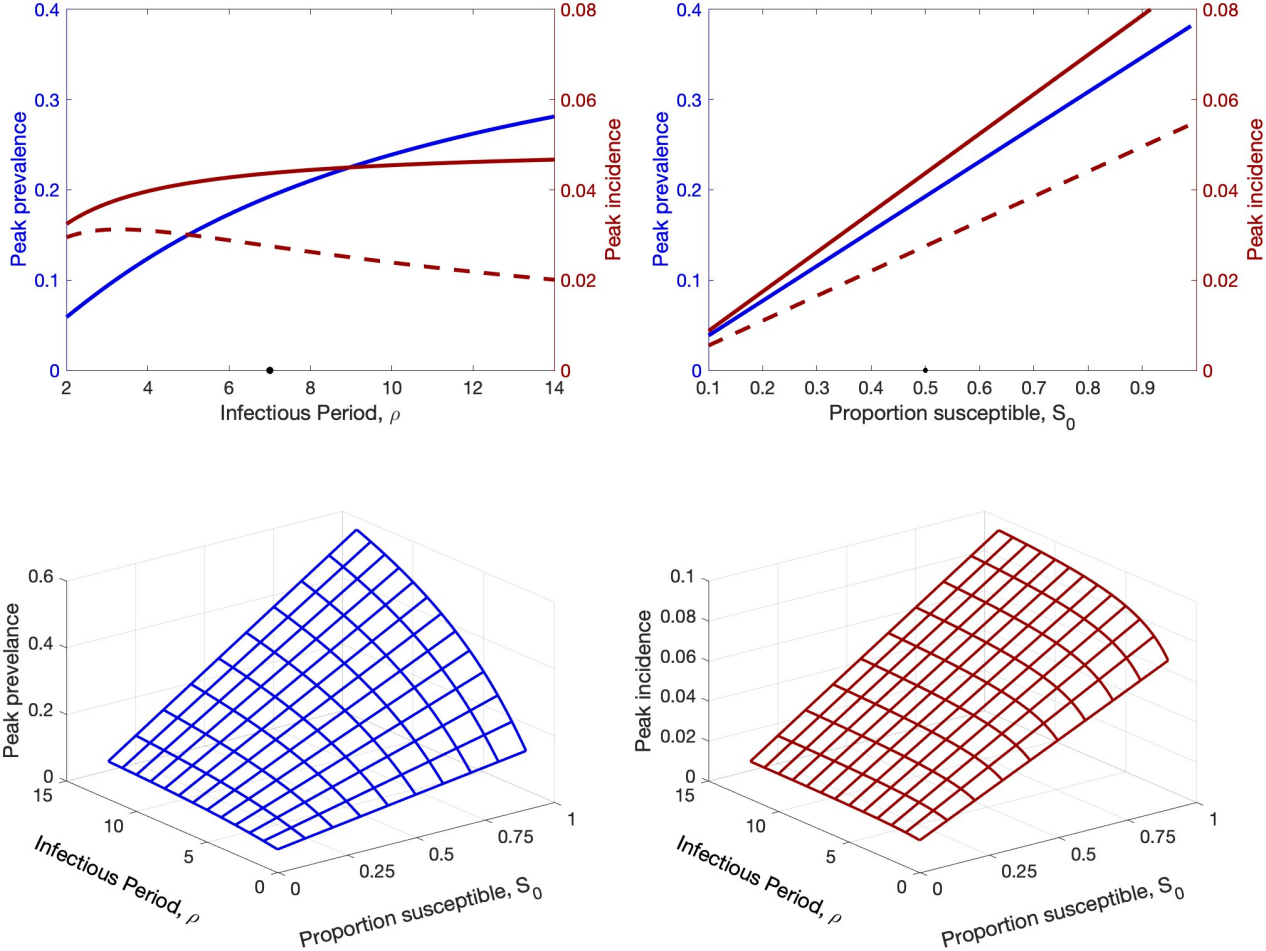
Results for the simple SIR model, (Eqs 2–4) and assuming a early growth rate of *r* = 0.4. Top panels show the peak prevalence, *I*_*p*_, (blue), the corresponding incidence *C*_*p*_ (dashed red) and true peak incidence *C*^*p*^ (solid red), as either the infectious period or the proportion of the population that are initially susceptible are varied. (Black dots on the x-axis show the default values *ρ* = 7 days and *S*_0_ = 0.5 used in the other graph.) Lower panels show the peak prevalence (*I*_*p*_) and peak incidence (*C*^*p*^) as both the infectious period and initial proportion susceptible vary.

At the peak prevalence of infection (when *dI*/*dt* = 0), we find that the level of susceptibles *S*_*p*_ = 1/*R*_0_, and can then use the standard Kermack and McKendrick [20] approach (which links *S*(*t*) and *R*(*t*) by considering $\frac{dS}{dR}$) to link the level of susceptibles (*S*_*p*_) and the level of recovereds (*R*_*p*_) at the peak:

$$S_{p}\;=\;S_{0}\;\text{exp}\;\left(-R_{0}R_{p}\right)\;\Rightarrow \;R_{p}\;=\;-\frac{1}{R_{0}}\;\text{log}\;\left(S_{p}/S_{0}\right),$$

in order to find the peak number of infections (*I*_*p*_). We do this by combining the facts that *S*_*p*_ + *I*_*p*_ + *R*_*p*_ = *S*_0_ and that *S*_*p*_ = 1/*R*_0_, together with writing *R*_0_ in terms of the growth rate (*R*_0_ = (*rρ* + 1)/*S*_0_) to find that:

$$\begin{array}{c} I_{p}\;=\;\frac{S_{0}}{r\rho +1}[r\rho -\text{log}\;(r\rho +1)], \end{array}$$ (2)

providing an estimate of peak prevalence in terms of the key parameters of the system.

While the peak prevalence is a natural quantity to calculate given the form of the SIR equations, for the majority of public health considerations it is more informative to consider the incidence or rate of generating new infections (*C* = *βSI*). At the peak in infections, this incidence is given by

$$\begin{array}{c} C_{p}\;=\;\beta S_{p}I_{p}\;=\;\frac{I_{p}}{\rho }\;=\;\frac{S_{0}}{r\rho^{2}+\rho }[r\rho -\text{log}\;(r\rho +1)]. \end{array}$$ (3)

The true maximum incidence, *C*^*p*^ occurs slightly earlier, but does not have a closed analytical form. It can, however, be expressed as the solution to:

$$\begin{array}{c} C^{p}\;=\;[\left(r\rho +1\right)S^{p}-S_{0}]\frac{S^{p}}{\rho S_{0}},\;\text{where}\;\;2\left(r\rho +1\right)\left(S^{p}/S_{0}\right)=\left(r\rho +2\right)+\text{log}\;\left(S^{p}/S_{0}\right). \end{array}$$ (4)

We note that in Eqs (2)–(4) the peak prevalence and peak incidence scale linearly with the initial proportion of the population that are susceptible (*S*_0_) (for Eq (4) the second expression implies that *S*^*p*^ is proportional to *S*_0_, hence the maximum incidence from the first expression is proportional to *S*_0_); however the behaviour with respect to *r* and *ρ* is non-linear. We expect this to transfer directly to the peak public health burden, which will also depend (linearly) on the infection to hospitalisation ratio, *μ*.

Fig 2 shows the computation of these peaks; highlighting that, for a fixed early growth rate (*r* = 0.4), the peak values vary linearly with the proportion of the population that are initially susceptible (*S*_0_) but non-linearly and saturating with the infectious period (*ρ*). This illustrates that from early epidemic data, when only the growth rate *r* is likely to be reliably estimated, it is difficult to predict the epidemic peak (whether we measure this as peak prevalence or peak incidence) due to its dependence on both the infectious period and the initial proportion of susceptibles. Moreover, in real-world scenarios (as shown below) there is the additional complication that only a proportion of infections are reported to become cases, and only a proportion of cases require medical attention; there is hence an additional proportionality constant (*μ*), which may be difficult to measure, that scales infections to the quantity we wish to consider.

### Fitting to a synthetic SIR epidemic

We now extend the analysis of the SIR model, by considering our ability to fit to the output of such simple models, treating it as synthetic data, and hence capture the peak hospital admissions generated by the model. We make use of a simple stochastic SIR epidemic (using the tau-leap method with Poisson distributions) and assume that the number of daily hospital admissions is a binomial distribution proportional to the daily recovery rate. More precisely:

$$\begin{array}{c} \begin{array}{ccccccc} {\text{Infection}}_{t} & = & \text{Poisson}(\beta S_{t}I_{t}/N) & \;\; & {\text{Recovery}}_{t} & = & \text{Poisson}(\gamma I_{t})\\ S_{t+1} & = & S_{t}-{\text{Infection}}_{t} & \; & I_{t+1} & = & I_{t}+{\text{Infection}}_{t}-{\text{Recovery}}_{t} \end{array}\\ R_{t+1}\;=\;R_{t}+{\text{Recovery}}_{t} \end{array}$$

$${\text{HospitalAdmissions}}_{t}=H_{t}=\text{Binomial}\left({\text{Recovery}}_{t},\mu \right)$$

In any stochastic simulation, there is inevitability considerable uncertainty when the number of infected individuals is low, leading to variablity in the timing of the peak. To normalise across multiple synthetics epidemics, the projected hospital admissions are therefore shifted in time such that the maximum of the seven-day moving average occurs at day 50.

In keeping with the model formulation used by the Warwick team throughout the COVID-19 outbreak; we fit a deterministic SIR model (Eq 1) to the hospital data using an MCMC (Markov Chain Monte Carlo) and assuming that observations are Poisson distributed with mean *h*_*t*_, proportional to the number of recent recoveries:

$$h_{t}=\mu \int_{-1}^{0}\gamma I\left(t+s\right)ds=\mu (R_{t}-R_{t-1}),$$

where *μ* is the constant of proportionality, also fitted in the MCMC process. This fitting procedure is performed on the synthetic hospital admissions data, up to time *T* and then the deterministic SIR model (with parameters drawn from the postior) is project forwards to determine the distribution of peak hospital admissions.

The log-likelihood of observing the synthetically generated number of hospital admissions up to time *T* from the deterministic model (with parameter set $\underline{\theta }=\left(r,\rho ,S_{0},\mu ,t_{0}\right)$) is therefore calculated as:

$${\text{LogLikelihood}}_{T}\left(\underline{\theta }\right)\;=\;\sum_{t=1}^{T}H_{t}\;\text{log}\;\left(h_{t}\right)-h_{t}-\text{log}\;\left(H_{t}!\right).$$

**Fig 3:**
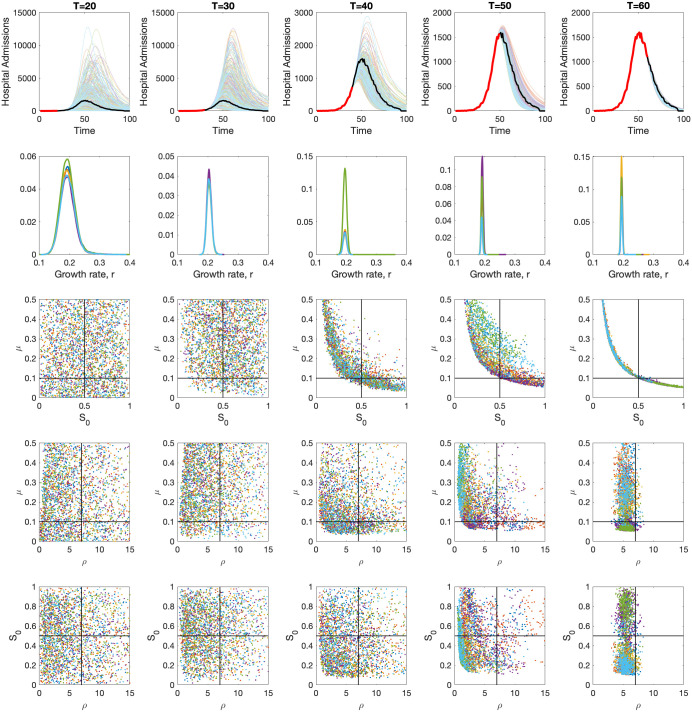
Progressive fits to the synthetic data for a population size *N* = 10^6^ at times *T* = 20, 30, 40, 50, and 60. The synthetic hospital admission data from one stochastic realisation is shown in the top row (red for the data used in the inference, black for the later data), together with the inferred deterministic levels of hospital admissions *h*_*t*_. The second row shows the inferred posterior distribution of the growth rate *r*. The lower three rows shows the correlation between the other three key parameters *ρ*, *S*_0_ and *μ* (the full range of *μ* and *ρ* values are not shown, instead we focus our axes on the main region of interest). All posteriors are samples from six independent chains (different colours), each with 10 million trial draws.

Fig 3 shows the outcome of fitting to progressively longer samples of an unfolding synthetic outbreak (*T* = 20, 30, 40, 50, 60). Table 1 gives the true values of the parameters used to generate the synthetic data and the assumed prior distributions (more informative priors would help in any practical situation). Early fits to the synthetic data (*T* ≤ 40) are unable to capture the future trends in numbers of hospital admissions and can only reliably capture one parameter: the growth rate *r* (Fig 3 second row). Later times show far better fits to the synthetic data, but there are still discrepancies between the true parameters and posterior samples. In particular, we note that there is a striking trade-off between *μ* and *S*_0_, such that only the product of the two can be determined—this conforms to the observation from simple theory that the peak number of hospital admissions is linear in both *S*_0_ and *μ* (see Simple Analysis above). We also observe that the inferred infectious period, *ρ*, is a very slight overestimate of the value used in the stochastic simulations, which we attribute to the use of a discrete time-step (tau-leap) methodology to generate the synthetic data in comparison to the continuous time deterministic model used in the fitting.

**Table 1:** Parameter values used in the synthetic stochastic SIR epidemic model and the assumed prior distribution. The value for *t*_0_ is not defined as the stochastic outputs are shifted to achieve the maximum number of hospital admissions at day 50.

Parameter	Meaning	Value	Prior
*r*	Growth rate	0.2	Γ(2, 0.1)
*ρ*	Infectious period	7	Exp(1/7)
*S*_0_	Proportion susceptible	0.5	*β*(1.5, 1.5)
*μ*	Infection hospitalisation ratio	0.1	*β*(1.05, 1.5)
*t*_0_	Initial time *S*(*t*_0_) = *S*_0_*N*, *I*(*t*_0_) = 1	-	*U*(−10, 0)

**Fig 4:**
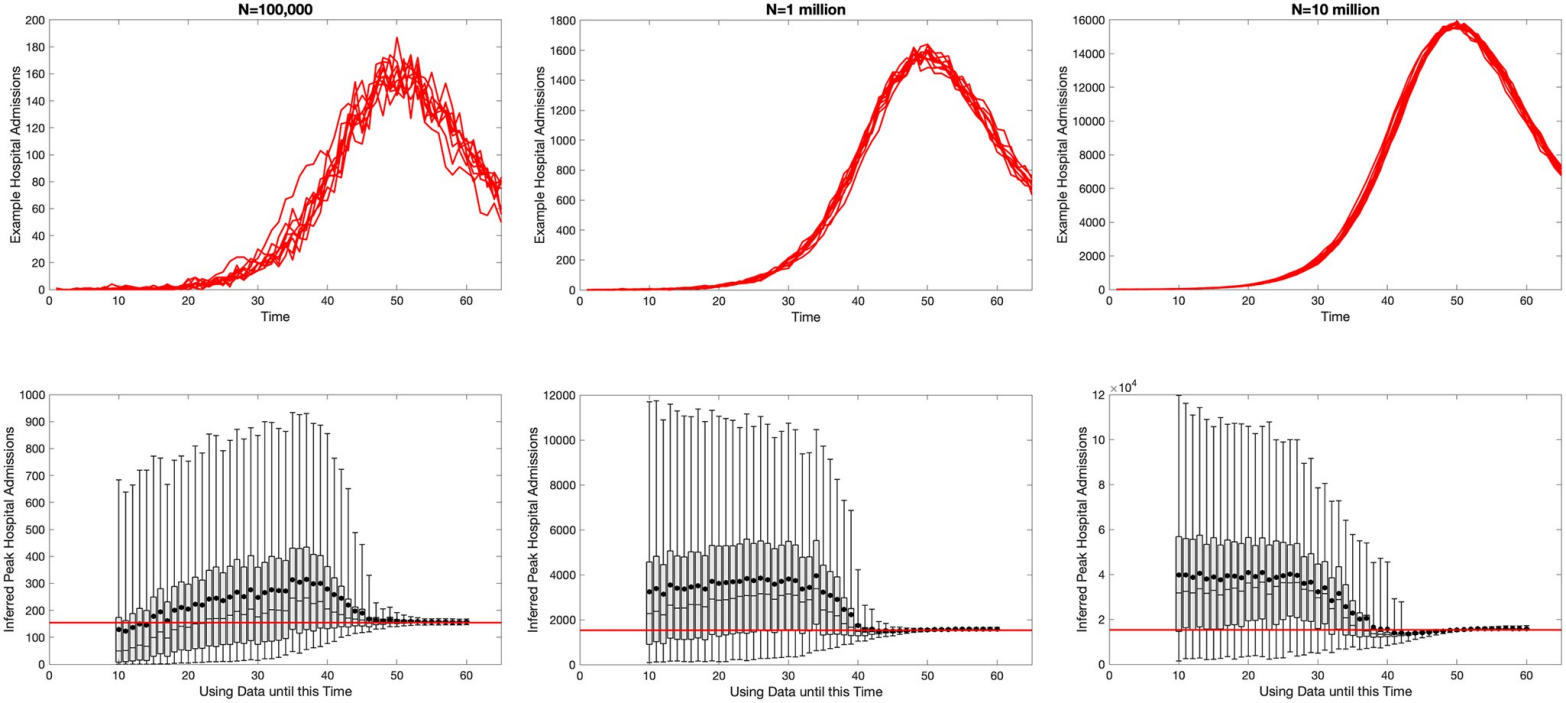
Ability of fitted SIR models to capture the peak number of hospital admissions (approximately 153 per 100,000 per day) for a synthetic population size of one hundred thousand (left), one million (center) and ten million (right). The top row shows ten replicates of the synthetic data for hospital admissions generated from the stochastic SIR model with parameters as in Table 1. The lower panels show the posterior distribution of predicted peak hospital admissions (*h*_*t*_), fit to a hundred stochastic replicates using a chain of length one million steps; the bar and whiskers show the interquartile and 95th percentile ranges as well as the median, while the dots show the mean value; the red horizontal line corresponds to the mean peak value.

Examining the fitting process at a finer temporal resolution, we consider the ability of the fitted model to capture the peak number of hospital admissions (Fig 4), and perform the fitting for multiple stochastic realisations of the underlying model. This agrees the findings in Fig 3; for small populations sizes of one hundred thousand there is an an inability to reliably predict the peak from early outbreak data and accurate projections only occur close to the peak (Fig 4, left-hand panels). For larger population sizes of one and ten million, our ability to predict the peak occurs from earlier time points (Fig 4, centre and right panels), but early projections still substantially over-estimate the peak number of hospital admissions.

Three linked facets combine to affect our ability to predict the peak: the stochasticity in the process generating the synthetic data; the uncertainty in the parameter estimates; and the non-linear dependence of the peak on these values. For small population sizes (*N* = 10^5^) and very early times (*T* < 15), we generally underestimate the peak, as the stuttering number of hospital admissions is insufficient to determine any of the parameters and *r* is often substantially underestimated [21]. However, once the dynamics have entered the phase of exponential growth, the projections generally overestimate the peak, both in terms of the projected mean and median; although the true values generally still well within the 95% prediction intervals. Examining the posterior distribution, we believe this over-estimation may be due to the choice of prior (the mean value of *μ* from the prior is larger that the value used); fixing *r* at its true value of 0.2 and picking other parameters from their prior distributions leads to an overestimate of the peak (median is approximately 233% of the synthetic value). We expect this observation to also hold early in an outbreak when there is limited data to inform parameter choices. (Interesting a more informed choice for *μ* ∼ *β*(1.05, 9.45) which has the same mean as used to generate the synthetic data, underestimates the peak—median is approximately 48% of the synthetic value, when *r* is fixed and we pick other parameters from the prior).

In theory, data from a deterministic model (or a very large stochastic population) should allow for the rapid estimation of all parameters, but as shown in Fig 4 even for population sizes of *N* = 10^6^ with relatively little stochastic noise, it is not feasible to reliably estimate parameters, and hence the peak, from early data. As illustrated in Fig 3, this parameter uncertainly only gets resolved once the outbreak approaches the peak; before this point, the interaction between the parameter uncertainty and the non-linear dynamics shown in Fig 2 leads to our inability to predict the peak.

### Analysis of the invasion of Omicron

We now switch our attention to the Omicron invasion and initially examine a proxy for the proportion of COVID-19 cases that are due to the Omicron variant, as this provides one of the earliest signals of growth of the new variant. This proxy is the proportion of TaqPath-tested PCR tests (with a *Ct* value below 30 such that there is sufficient virus in the sample), that are negative for the S-gene [22, 23]; the BA.1 variant of Omicron is S-gene negative, whereas BA.2 (which followed the BA.1 wave) and Delta (which preceded the BA.1 wave) are both S-gene positive. While this proxy is not as sensitive nor as specific as the full genomic sequencing that was additionally performed [6], it has the benefits of generating more rapid results and having a larger sample size (e.g. on 15th December 2021 there were around 29,000 samples sequenced, but 64,000 positive samples that were TaqPath-tested).

To examine the data from the early invasion of Omicron, we utilise a standard SIR-type model with two competing pathogens (in this case *I*_1_ for Delta and *I*_2_ for Omicron, BA.1):

$$\begin{array}{ccc} \frac{dS}{dt} & = & -\beta_{1}SI_{1}-\beta_{2}SI_{2}\\ \frac{dI_{1}}{dt} & = & \beta_{1}SI_{1}\;-\;\gamma_{1}I_{1}\\ \frac{dI_{2}}{dt} & = & \beta_{2}SI_{2}\;-\;\gamma_{2}I_{2} \end{array}$$ (5)

We find that the proportion of infections that are Omicron (labelled *p* = *I*_2_/(*I*_1_ + *I*_2_)) should follow a sigmoidal growth curve:

$$\begin{array}{ccc} \frac{dp}{dt} & = & \frac{d}{dt}\;\frac{I_{2}}{I_{1}+I_{2}}\;=\;[\left(\beta_{2}S-\gamma_{2}\right)-\left(\beta_{1}S-\gamma_{1}\right)]p\left(1-p\right)\\ \Rightarrow \;p(t) & = & \frac{\text{exp}\;(\left[r_{2}-r_{1}\right]\left(t-\tau \right))}{1+\text{exp}\;(\left[r_{2}-r_{1}\right]\left(t-\tau \right))} \end{array}$$ (6)

where *r*_1_ and *r*_2_ are the relative growth rates of the two pathogens (*r*_*i*_ = *β*_*i*_*S* − *γ*_*i*_). If the level of susceptibles is not substantially depleted during the invasion phase, then the difference in growth rates (*r*_2_ − *r*_1_) can be assumed constant. In Eq (6), the parameter *τ* is a constant that is determined by the initial level of the invader, and is also the inflection point of the sigmoidal curve, corresponding to when *p*(*τ*) = 0.5.

**Fig 5:**
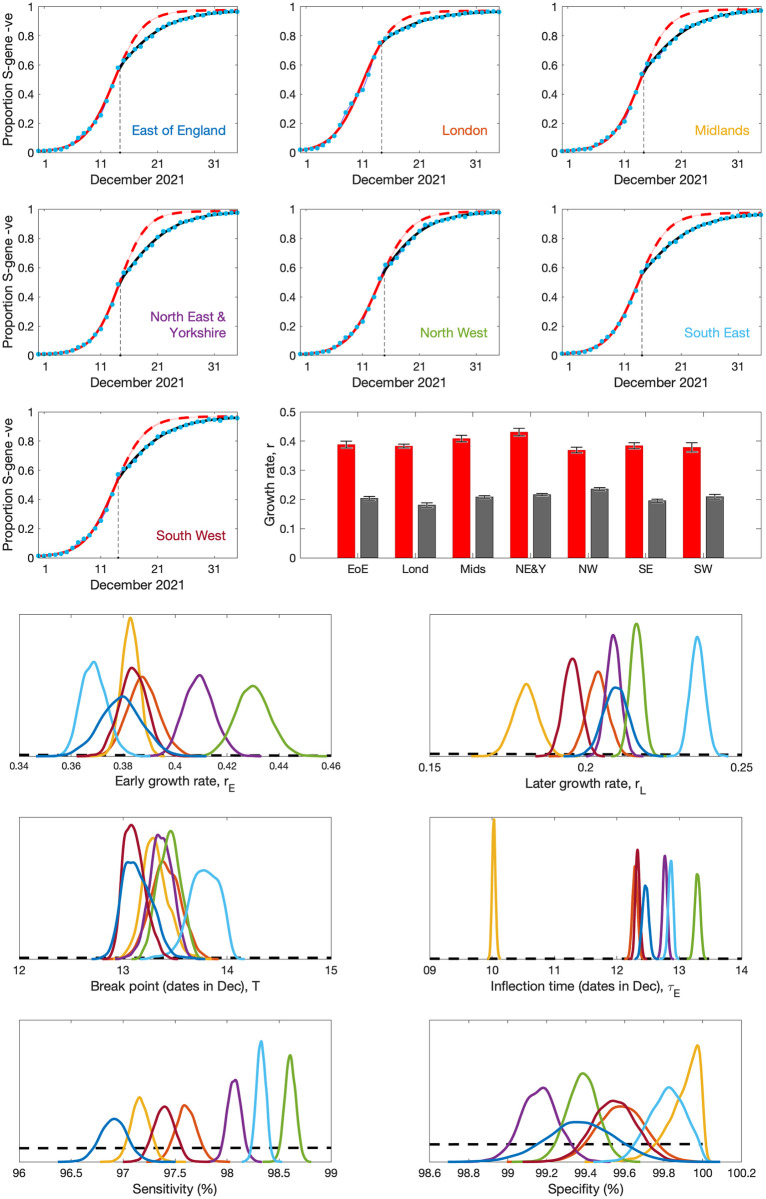
Analysis of the invasion of the Omicron variant through December 2021. The top panels show the rise of the proportion of positive PCR tests that are S-gene negative (a proxy for Omicron infection); blue dots are the data for each of the seven NHS regions, red curves are a sigmoidal fit (using maximum likelihood) to the early data until 13th December, while black curves are fits to the later data only. The estimated early (red) and late (black) sigmoidal growth rates (*r*_*E*_ and *r*_*L*_) are shown in the central bar-plot, with error-bars capturing 95% credible intervals. The lower six panels show the posterior distributions of parameters from MCMC fitting performed on the same data sets; together with the prior distributions (black dashed line) which are only weakly informative. Distributions are colour-coded as used in the names on the top panel: East of England—blue; London—red; Midlands—orange; North East and Yorkshire—purple; North West—green; South East—light blue; South West—dark red.

Considering the COVID-19 case data from England, we observe that the daily proportion of positive tests performed on the TaqPath system that are S-gene negative (and therefore likely to be Omicron) generally follows a sigmoidal trend (Fig 5) as predicted by the simple SIR model. However, closer inspection shows that there are different sigmoidal growth rates in early and late December with the later period associated with a slower growth. Using S-gene data from each of the seven NHS regions in England (East of England, London, Midlands, North East and Yorkshire, North West, South East and South West), we fit the invasion with two sigmoidal curves for the early and late behaviour:

$$P\left(t\right)=\{\begin{array}{cc} \varepsilon \;+\;\left(1-\varepsilon -\hat{\varepsilon }\;\right)\frac{\text{exp}\;(r_{E}\left(t-\tau_{E}\right))}{1+\text{exp}\;(r_{E}\left(t-\tau_{E}\right))}\; & \text{if}\;t<T,\\ \varepsilon \;+\;\left(1-\varepsilon -\hat{\varepsilon }\;\right)\frac{\text{exp}\;(r_{L}\left(t-\tau_{L}\right))}{1+\text{exp}\;(r_{L}\left(t-\tau_{L}\right))}\; & \text{otherwise}, \end{array}$$

where *r*_*E*_ and *r*_*L*_ are early and late growth sigmoidal rates, *T* is the time point separating the two growth regimes, *τ*_*E*_ is the early inflection point and *τ*_*L*_ is the late inflection point (which is determined by the other parameters to ensure continuity of the curve at *T*). The fit also includes the possibility of false positive and false negative results, as such $\left(1-\hat{\varepsilon }\;\right)$ is the sensitivity of an S-gene negative result for predicting Omicron infection and (1 − *ε*) is the associated specificity. MCMC parameter inference (with the likelihood determined by assuming tests are binomially distributed) is used throughout to gain an understanding of parameter uncertainty. This echoes the approach of [24].

The red lines on the upper panels of Fig 5 illustrate that the early dynamics, for the first half of December 2021, are well captured by a sigmoidal curve in which the estimated sigmoidal growth rate *r*_*E*_ is ∼ 0.4 per day (Fig 5 bar graph). From the simple 2-species SIR model (Eq 5), this means that the growth rate per day of Omicron (*r*_*Omicron*_) is around 0.4 per day higher than *r*_*Delta*_, and given that the Delta variant maintained a relatively constant level of infection throughout September-November 2021 we can assume *r*_*Delta*_ ∼ 0. This supports the value of *r* = 0.4 assumed in Fig 2, and would suggest an early doubling time of the Omicron wave of around 2 days. However, from mid-December, there is a pronounced departure from the earlier sigmoidal trend (shown with a dashed line in Fig 5), and a new sigmoidal pattern with a lower difference in growth rates (∼ 0.2 per day) is a better fit. The early and late sigmoidal growth rates (*r*_*E*_ and *r*_*L*_, estimated by maximum likelihood) for each of the seven NHS regions (Fig 5, centre right bar graph) clearly demonstrate the scale of this change. We note that for the default epidemiological parameters used in Fig 2 (*ρ* = 7 days, *S*_0_ = 0.5) an *r* ∼ 0.4 growth rate leads to a peak infection rate of *C*^*p*^ ≈ 4.4% of the population, whereas when *r* drops to around 0.2 the peak infection rate is estimated at *C*^*p*^ ≈ 1.9% of the population. For all seven NHS regions in England, the break point between early and late growth rates is estimated to be around 13th December. The consistency of this date between regions, together with the dramatic change in the sigmoidal growth at this time, suggests a sudden change in national behaviour—reducing the relatively growth rate of Omicron compared to Delta.

The optimal model, in terms of minimising the number of parameters but still obtaining a good fit, is achieved when there is a single break point parameter but all other parameters are specific for the region; this can be formally captured using information criterion measures. In Table 2 we report the Bayesian Information Criterion [25], which seeks to balance model fit and the number of parameters estimated. We consider 16 models, with different patterns of uniform (same parameter for each region) and regionally specific parameters, and report the number of fitted parameters and the BIC relative to the model shown in the main paper where each region has its own set of independent parameters. The only model that has an improved BIC compared to the full model (where all parameters are regional) is when the break time associated with the change in *r* is the same for all regions.

**Table 2:** Relative Bayesian Information Criterion of 16 models compared to the full model where all parameters are regional. We also include the number of parameters that are inferred for each model.

Description of model parameter sets	Number of parameters	Δ BIC
All parameters regional	36	0
Uniform infection time	31	4467.14
Uniform sensitivity	31	6.24
Uniform specificity	31	329.70
Uniform early growth	31	46.35
Uniform later growth	31	195.43
Uniform break time	31	**-16.66**
Uniform sensitivity and specificity	26	338.61
Uniform early and later growth	26	242.76
Regional infection times	11	3221.71
Regional sensitivities	11	13784.30
Regional specificities	11	18666.30
Regional early growth rates	11	8982.03
Regional later growth rates	11	15141.90
Regional break times	11	9685.34
All parameters uniform	6	20672.50

Examining the posterior distributions in more detail we find that all distributions are relatively tight and have significantly departed from the prior distributions. Early growth rates range from 0.35 to 0.45 per day, while later growth rates range from 0.17 to 0.25 per day; it is interesting to note that the North West of England (shown in green) has the slowest early growth rate but the fastest later growth rate. Break points in all regions are extremely tightly clustered during 13th December. The inflection time (*τ*_*E*_) shows much more variability between regions, likely due to the much earlier arrival of Omicron in London compared to the rest of England giving it an inflection time over two days (one doubling period) earlier. Finally, sensitivity and specificity are both high (sensitivity in the range 96.5-99%, specificity in the range 98.9-100%) although there is considerably variability between regions, potentially due to the presence of other variants at relatively low levels.

### Predictive modelling of Omicron

We now consider a predictive mathematical model used during the Omicron outbreak [11], and highlight our ability (or inability) to accurately predict the likely scale of the Omicron wave. Two inference procedures are considered, both operate at the scale of NHS regions and match to the proportion of PCR tests that are positive cases, the proportion of cases that are S-gene negative, the number of daily hospital admissions, the hospital occupancy and the daily mortality within 28 days of a positive COVID-19 test; this fitting procedure has been described in detail in [26] and subsequent work [27, 28]. In our first inference process, which is most appropriate for the earliest stage of the outbreak, Omicron is assumed to have similar characteristics to the dominant Delta variant, but with a higher transmission rate. This inference is driven primarily by the growth rate of Omicron (or more precisely the growth rate of Omicron relative to Delta) and is therefore comparable to that used in Fig 5. Given only a single new parameter is involved, the inference procedure can be performed with relatively limited early data. The second approach also infers the duration of infection, the case hospitalisation ratio and the level of immune escape, in addition to a transmission rate for Omicron.

For both inference approaches, there is an additional precautionary behaviour parameter which changes gradually each week (by no more than 0.2); this parameter captures a range of human behavioural changes (such as lockdowns, increased mask use or greater compliance with test-and-trace) in a single unifying parameter which then impacts the transmission rate of all variants equally. The fitting to the Omicron epidemic benefits enormously from the statistical estimates of vaccine efficacy, vaccine waning and the impact of the booster dose [29] that were performed in early December 2021. This additional information reduced the number of parameters that need to be estimated, and shows how protection against Omicron was far lower than Delta and waned to almost zero by 25 weeks after the second dose (SI).

**Fig 6:**
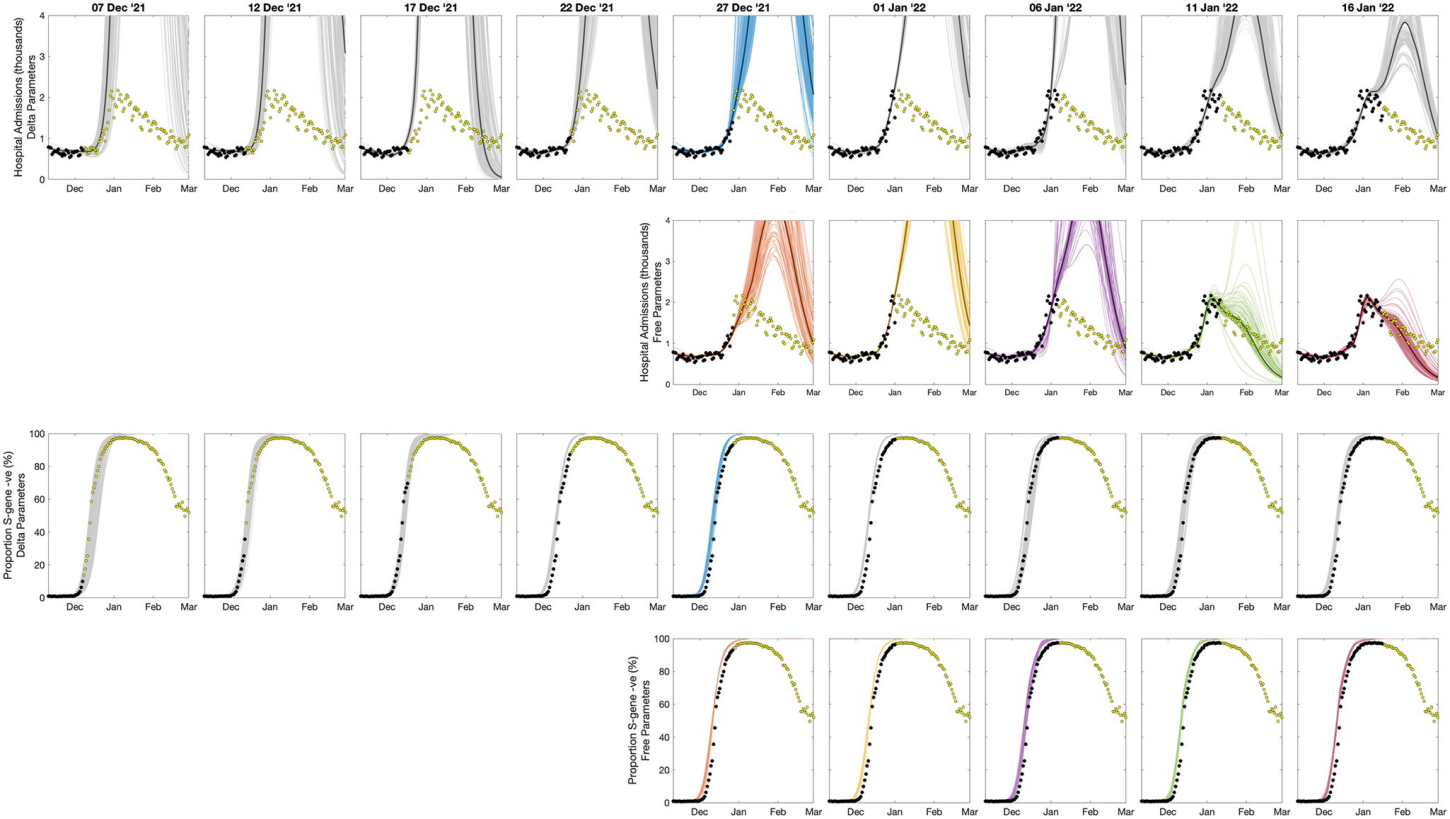
Progressive fits to the epidemiological data, showing data (dots) and predictions (lines) of daily hospital admissions (rows 1 and 2) and the proportion of S-gene negative PCR tests (rows 3 and 4) for the whole of England. The model is fitted to observed infections, hospital admissions, deaths and S-gene results in each of the seven NHS regions and projections are amalgamated to give the national pattern. The data points used in fitting the model are shown in black, with later data in yellow; 100 samples from the posterior projections are shown as thin lines while the median is shown as a bold darker line. Rows 1 and 3 assume that the Omicron variant has the same epidemiological parameters as Delta but with a higher (estimated) transmission rate, while in rows 2 and 4 all epidemiological parameters associated with Omicron are estimated. Coloured projections (Delta-parameters using data from 27th December 2021, and fully estimated parameters from 27th December onward) correspond to parameters in Fig 7.

We perform the two parameter inference schemes every five days from 7th December 2021 to 16th January 2022, using complete data records from England; although we note that at the time, due to the Christmas holidays, data was unavailable from 18th December to 5th January. In addition, there was generally a few days delay between some events and the data becoming available (e.g. between testing, being identified as positive and the data being collated). Therefore, the refitting of the model is somewhat of an idealised system, but still highlights the challenges of fitting a rapidly growing epidemic in real time. We show two outputs from the fitting and simulation procedure: the number of predicted daily hospital admissions in England (Fig 6, rows 1 and 2); and the proportion of TaqPath PCR tests that are S-gene negative—a proxy for the proportion of cases due to the Omicron variant (Fig 6, rows 3 and 4). These results are shown for both the most parsimonious inference mechanism where only the transmission rate of Omicron is allowed to vary, with all other parameters taking the same values as the Delta variant (rows 1 and 3) and the full inference model where all Omicron variant parameters are allowed to vary (rows 2 and 4).

**Fig 7:**
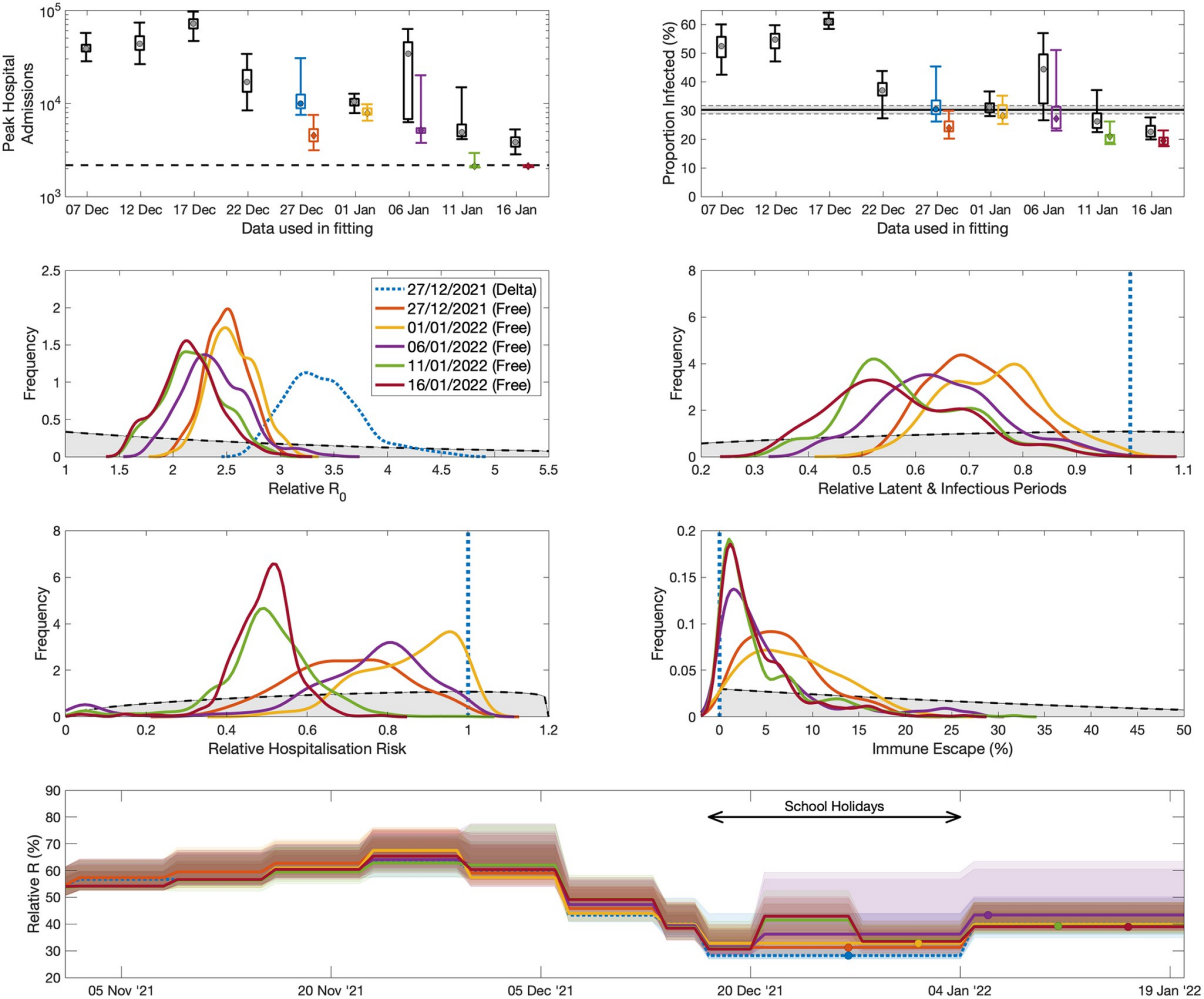
Progressive fits to the epidemiological data, showing two key model outputs (top row) and posterior distributions of inferred parameters (rows 2-4); colours correspond to those in Fig 6. Top left: projected peak daily hospital admissions from models matched to data up to various time points (as in Fig 6), showing 95% and 50% prediction intervals (bar and whisker plots) together with median values (dots), the dashed line shows the true value—note results are plotted on a log scale. Top right: projected proportion of the entire population of England infected in the Omicron wave (1st Dec 2021 to 11th February 2022), the horizontal line is the estimate from the ONS COVID-19 survey [30]. The central four panels show the posterior distributions for the relative *R*_0_, the relative duration of latent and infectious periods, and the relative hospitalisation risk (all compared to inferred values for Delta) and the level of immune escape for Omicron; the shaded grey area shows the prior distributions. The lower panel shows the inferred reduction in reproductive ratio due to changes in population-level mixing (compared to pre-COVID mixing patterns); this reduction includes levels of voluntary or enforced precautionary behaviour and school holidays but ignores the depletion of susceptibles over time.

Early projections, assuming Omicron parameters are the same as those for the Delta variant, fitted to observations up to 1st January 2022 are a good fit to the available data (shown as black dots) for the number of hospital admissions (Fig 6, top row) and capture the growth of Omicron (S-gene negative PCR samples) relative to Delta (S-gene positive samples) (Fig 6, third row). Driven by the rapid growth of Omicron (relative to Delta), these early projections lead to a massive wave of hospital admissions in January 2022 (see also Fig 7 top left). This closely corresponds to the simple system envisaged in Fig 2 where, for *r* = 0.4 and default parameters, the infectious wave peaks with *C*^*p*^ ≈ 4% of the population newly infected in a single day. The peak levels of infection in the COVID-19 model are lower than this theoretical value (estimated to peak at around 1-2%) due to the action of heterogeneous age-structured mixing within the more complex model.

From late December there is sufficient data to attempt to infer the complete range of Omicron parameters (projections shown in Fig 6 rows 2 and 4; inferred parameters in Fig 7), although we also continue to fit the more parsimonious model. We terminate the fitting process in mid-January 2022, by which time there has been an obvious turn-over in hospital admissions and the proportion of S-gene negative tests has begun to decline due to the invasion of the S-gene positive Omicron BA.2 strain. The parsimonious model continues to predict a substantial peak in hospital admissions even in mid-January driven by the observed growth advantage of Omicron; however it is only by 6th January that there is a noticeable difference between the hospital admissions data and the projections—although, arguably this could have been driven by extreme changes in mixing behaviour over the Christmas and New Year period. The full inference process leads to projected peaks that decline as more data is accumulated (see also Fig 7), however it is only on 11th January 2022 that there is sufficient weight of data that the inferred hospitalisation risk declines and the majority of projections capture the true peak. By 16th January, all (full inference) projections are correctly capturing the peak. This later model predicts a continual decline in hospital admissions during January and February 2022, this is prevented by the invasion and increase of Omicron BA.2 cases.

Considering aggregate measures of the model projections (Fig 7 top row), we can quantify the accuracy of the fitting and projection procedures. Using data up to mid-December, and assuming Delta-like parameters, the model projections describe an outbreak with extremely high peak hospital admissions in excess of 20,000 per day, and an outbreak that infects over 50% of the population. The data from 22nd December leads to a decline in both measures, with this decline continuing as more data is accrued, although the parsimonious model always substantially over-estimates the peak of hospital admissions. Later fits slightly underestimate the number of infections in the Omicron wave (1st December 2021 to 11th February 2022) compared to ONS survey estimates (shows as a horizontal lines). We postulate that this may reflect our inability to capture the extreme behavioural changes over the Christmas period when both health-seeking and testing behaviour may be substantially different from the norm.

The central two rows of Fig 7 show the posterior distributions inferred for some of the key Omicron parameters relative to the parameters for Delta. The parsimonious inference process (blue dashed line) leads to a substantial increase in transmission and hence *R*_0_ for Omicron compared to the Delta variant given that all the other parameters are assumed to be the same for Delta and Omicron. When parameters are free to vary (solid lines) we note that there is a reduction in the duration of the latent and infectious periods compared to Delta and consequently *R*_0_ does not have to be so large to achieve the same early growth rate. As more data is used in the inference process, the risk of hospitalisation drops to around half that of Delta, while there is an increased risk of immune escape such that previous infection with a former variant (Wildtype, Alpha or Delta) does not provide complete immunity against the Omicron variant.

Finally, we consider the impact of the inferred level of precautionary behaviour (Fig 7, lower panel). Here we have converted precautionary behaviour (which is an arbitrary measurement, see Methods) into the relative value of *R* (ignoring the depletion of susceptibles) under this behaviour compared to pre-COVID-19 mixing. Throughout November, we infer that the level of precautionary behaviour declines and therefore *R* would increase (ignoring the impacts of vaccination and depletion of susceptibles). From early December onward we infer a decline in *R* due to human behaviour from a maximum of around 65% to a minimum of around 35% (compared to the *R* value associated with pre-COVID levels of mixing). This halving of transmission, corresponds well with the sigmoidal fitting (Fig 5) where again the growth rate approximately halved. However, while the sigmoidal fit is constrained to change at a single time point, the precautionary behaviour is constrained to change gradually each week to prevent over-fitting, which may in part explain some of the differences. Without this increase in precautionary behaviour and the associated decline in mixing, the final epidemiological parameter estimates for Omicron (taken from 16th January 2022) would still lead to a large outbreak of around 27,000 (95% CI 20,000-44,000).

## Discussion

Here we have considered four approaches that provide generic and specific insights into fitting to an outbreak in real-time.

The analytical results for the simple SIR model highlight that for a given growth rate (which we assume we can robustly infer from the data) the peak prevalence and incidence both scale linearly with the proportion of the population that are susceptible to the infection, but scale non-linearly with the infectious period. If the quantity of interest is more severe infections (such as those requiring hospital treatment) then this places an additional linear scaling on the predicted peaks.

This simple SIR approach is then extended by inferring parameters from the early stages of an outbreak and thereby predicting the peak level of hospital admissions. This approach shows surprising correspondence with the inference for Omicron, which is based on a far more complex model and a richer data set. In particular, when faced with multi-dimensional parameter uncertainty, it is extremely difficult to predict the peak until there has been a substantial reduction in the rate of exponential growth and the system is approaching the peak (Fig 4). In addition, for this simple approach the values of the proportion of the population that are susceptible and the infection hospitalisation ratio (*S*_0_ and *μ*) cannot be uniquely determined (Fig 3). In contrast, the results for the Omicron models have the advantage of being matched to multiple data sources (proportion of tests that are positive, proportion of positive tests that are S-gene negative, number of hospital admissions, hospital occupancy and number of daily deaths), potentially increasing parameter identifiablity. However, compared to the synthetic results from an SIR model, the data from the Omicron BA.1 wave is also more complex including age and spatial heterogeneities as well as temporally varying mixing patterns.

In the sigmoidal fit (Fig 5) we constrained the transmission rate to change at a single point in time, corresponding to an abrupt switch in the level of precautionary behaviour. In all seven English NHS regions, this change point occurred on 13th December (with very little variation when using MCMC), and reduced the sigmoidal growth rate of Omicron by around 50%—comparable to the reduction seen in the full model between late-November and mid-December (Fig 7). The cause (or causes) of this change is unclear and cannot be inferred from the data. The move to ‘Plan B’ occurred on 8th December 2021 and involved working from home where feasible, the use of face masks in most public indoor venues, daily tests for contacts of confirmed positive cases (instead of the ten-day self-isolation period [31]) and recommendations for increased LFD testing especially when visiting vulnerable individuals [32]. This message on increased testing was re-enforced during much of December; reported cases identified through LFD testing increased dramatically over this period, from around 1300 on 1st December to a peak of nearly 20,000 on 29th December, presumably reflecting an increase in LFD testing and therefore leading to a decline in transmission. The growth of cases will also be restricted by the school holidays and reduced working over the Christmas period.

Considering the fitting and projection of the full model (Figs 6 and 7) to the sequential data throughout the Omicron wave provides some insights into the scientific evidence presented during December 2021 [11]. In the absence of data about the distribution of incubation and infectious periods, the natural assumption was that these were broadly comparable to those measured for Delta and previous variants. This assumption coupled with the rapid growth of Omicron (either measured through S-gene failures in the TaqPath system or through genomic investigation [6]), suggested that Omicron had a far larger basic reproductive ratio (*R*_0_) and would therefore peak at very high levels of infection (and correspondingly high levels of hospital admissions). It is not until early January that there is any indication that the aggregate pattern of cases and hospital admissions deviates from the projections which assume that Omicron has similar characteristics to Delta (Fig 6, rows 1 and 3). Including a lower severity as potentially indicated by South African [13] or preliminary UK data [14] would introduce a multiplicative scaling to the projections, but this scaling factor is insufficient to overcome the early over-estimation of peak.

In principle the Omicron outbreak in England should have been an idealised example of real-time model projection: there were a range of well-calibrated models that had been improved and matched to data throughout the pandemic [27, 33–37]; there were nation-wide approaches to collect and collate daily data on tests, cases, hospital admissions and deaths; and the TaqPath PCR S-gene data and sequencing data had provided a clear quantification of the growth of Omicron relative to the resident Delta variant.

However, Omicron represented a distinct parameter shift compared to previous variants; Wild type, Alpha and Delta all had comparable incubation and infectious periods [16, 17], and there had been a notable increase in the risk of hospital admission over time from Wild type to Alpha to Delta. Yet our estimates for Omicron had a faster generation time and a lower risk of severe symptoms (Fig 7), reversing the previously observed trend. To identify and capture this parameter shift requires substantial amounts of data; the drop in hospitalisation risk is not captured by our inference mechanisms until data from 11th January 2022 is available. In principle, identifying the variant leading to hospital admissions could have more rapidly indicated the lower hospitalisation risk [14]. Some of the early data from South Africa did point to a lower risk of severe illness, but due to differences in epidemiological history and population age-structure between the UK and South Africa it was dangerous to assume that the observations would directly translate. In particular, the substantial increase in vaccinated individuals in South Africa prior to the Omicron wave complicated the comparison of hospital admissions with the previous Delta wave, particularly in the absence of data reporting the vaccination status of hospital admissions. The generation time, incubation and infectious periods for Omicron have been estimated by a number of authors, although conclusions are conflicted and great statistical care is required: shorter periods have been reported [38, 39], as have shorter generation times [40]; while others suggested that incubation periods, serial intervals and instrinsic generation times are similar Omicron BA.1 and Delta variants [41, 42]. This strongly suggests that the change in hospitalisation risk and time-scales of Omicron BA.1 compared to Delta require data from an outbreak that is approaching its peak. This is supported by our inference of the simple SIR model 3, where parameters other than *r* are not reliably determined until close to the peak.

The rapid replacement of the Delta variant by the Omicron variant during December 2021, pointed to a substantial transmission advantage, with Omicron cases doubling every 2 days compared to a roughly constant number of Delta cases 5 [24]. This would naturally lead to predictions of a large peak in infection and therefore a large peak in hospital admissions. Model projections from early December suggested a peak of around 7500 daily hospital admissions (in a worst case scenario, but with additional mask wearing and Plan B restrictions [12]) or over 20,000 daily hospital admissions (in a worse-case scenario without controls, [11]). These extremely high values are in keeping with the model fits to early December data (Fig 7) when Omicron is assumed to have Delta-like characteristics. Four factors acted to reduce the peak in hospital admissions from these initially high projections:

- First is the slightly shorter duration of the incubation and infectious periods (Fig 7) meaning that there is a lower *R*_0_ associated with Omicron for the same observed growth rate. This lower *R*_0_ means that the peak of infection occurs at a lower level of susceptibles, this occurs earlier in the epidemic wave and therefore at a lower number of infected individuals.
- Secondly, the lower severity of Omicron leading to a lower risk of hospital admissions per infection and therefore a proportionate reduction in the peak of hospital admissions. Our final estimated level of severity of 49.3% (CI 33.8-63.5%, Fig 7) compared to the Delta variant is remarkably close to the early estimates of 55% (CI 51-59%) based on all reported cases and hospitalisations of at least one day [14]. This estimate is notably higher than 20% (CI 10-30%) figure from South Africa [13], which may be attributable to very different population demographics and history of vaccine uptake between the two countries.
- The third factor reducing the peak is the increased uptake of the booster vaccine during December, which provides strong short term protection against hospitalisation with Omicron even in those individuals whose protection against infection had effectively waned by this time [29, 43]. Booster uptake in November 2021 was around 286,000 per day (with a peak on 430,000), however during early December the number of boosters administered peaked at over 850,000 per day.
- The final, and arguably most important, contributing factor is the change in human behaviour during December. In our predictive model fit to the Omicron BA.1 wave (Fig 7) this appeared as a drop in the reproductive ratio throughout December, while in our analysis of the Omicron invasion (Fig 3) this is realised as a step change on 13th December. Although the elements of this change cannot be determined by our inference process, the regional synchrony of the changes suggests three potential national-scale contributing factors: the implementation of Plan B restrictions on 8th December, including compulsory face masks in many settings, increased working from home where possible and mandatory NHS Covid Pass (proof of vaccination status or proof of negative test) in specific settings; the increased awareness of the risks from the Omicron outbreak leading to greater amounts of testing especially before mixing with vulnerable people; and the general reduction in work-based mixing over the Christmas period including the reduced mixing of pupils during the school holidays. This is supported by both the ONS Opinions and Lifestyle Survey results showing increased testing and increased in mask use (see Fig 1 from [44]) during December 2021 and January 2022, and a reduction in mean contacts over the Christmas period estimated by Comix ([45]).

Multiple lessons can be learned from the real-time modelling of the Omicron BA.1 wave based on our retrospective analysis. The first is that robust modelling and parameter inference takes time—both in terms of data requirements and also in terms of fully exploring all uncertainties. Omicron BA.1 was first reported in the UK on 27th November 2021, and started to increase rapidly out-competing the resident Delta variant. The observed rapid increase placed substantial pressure on the involved modelling teams to generate projections. These projections, first generated in early December [7, 10] were largely based on the observed growth rate of Omicron relative to Delta, and had to make educated assessments of the impact of policy changes due to Plan B or behavioural changes over the Christmas period; yet experience with the 2021 road-maps for relaxation of controls showed that at least 4-weeks were required to determine the impact of changes to controls [27]. Our second lesson is that while early models did consider numerous uncertainties (including disease severity, generation time and population behaviour) without evidence to support alternative assumptions such sensitivity analyses were largely supplementary and not fully reflected in the main results—even though with hindsight some of these scenarios closely matched observations. For these policy-relevant projections a careful balance needs to be achieved between including enough uncertainties that policy-advisers understand the range of outcomes, and not including so many uncertainties that the results have no predictive merit. Our final conclusion is the difficulties of accounting for human behavioural patterns [46–49], not only in terms of reaction to public health messaging and epidemiological threats but also how social and work interactions changed over the Christmas period. Projections over this period were also exacerbated by the Christmas break, due to a lack of regular data updates through the normal automated channels from 18th December 2021 to 5th January 2022.

We therefore conclude that robustly predicting the peak number of hospital admissions from the Omicron wave was impractical from the data available in early December. However, the results presented to SAGE [7, 8] were pragmatic and precautionary—in terms of making minimal assumptions in the context of considerable uncertainty. The early Omicron projections showed considerable short-term accuracy (only deviating from observations in early January), while some of the scenarios investigated show long-term agreement (Fig 1). However, this does not imply that such real-time projections are not an important public health tool. With better quantitative understanding of key epidemiological parameters (e.g. infection hospitalisation ratios, latent and infectious periods) and with a less unpredictable behavioural context, our work has demonstrated that projections can be robust. Moreover, even in settings that lead to considerable uncertainty, knowing that there are a range of plausible outcomes can be hugely significant.

## Methods

Although the development of the Warwick SARS-CoV-2 transmission and COVID-19 disease model has been described elsewhere in extensive detail [26, 33, 50, 51], here we summarise the main salient components and the method of parameter inference.

### Model overview

**Fig 8:**
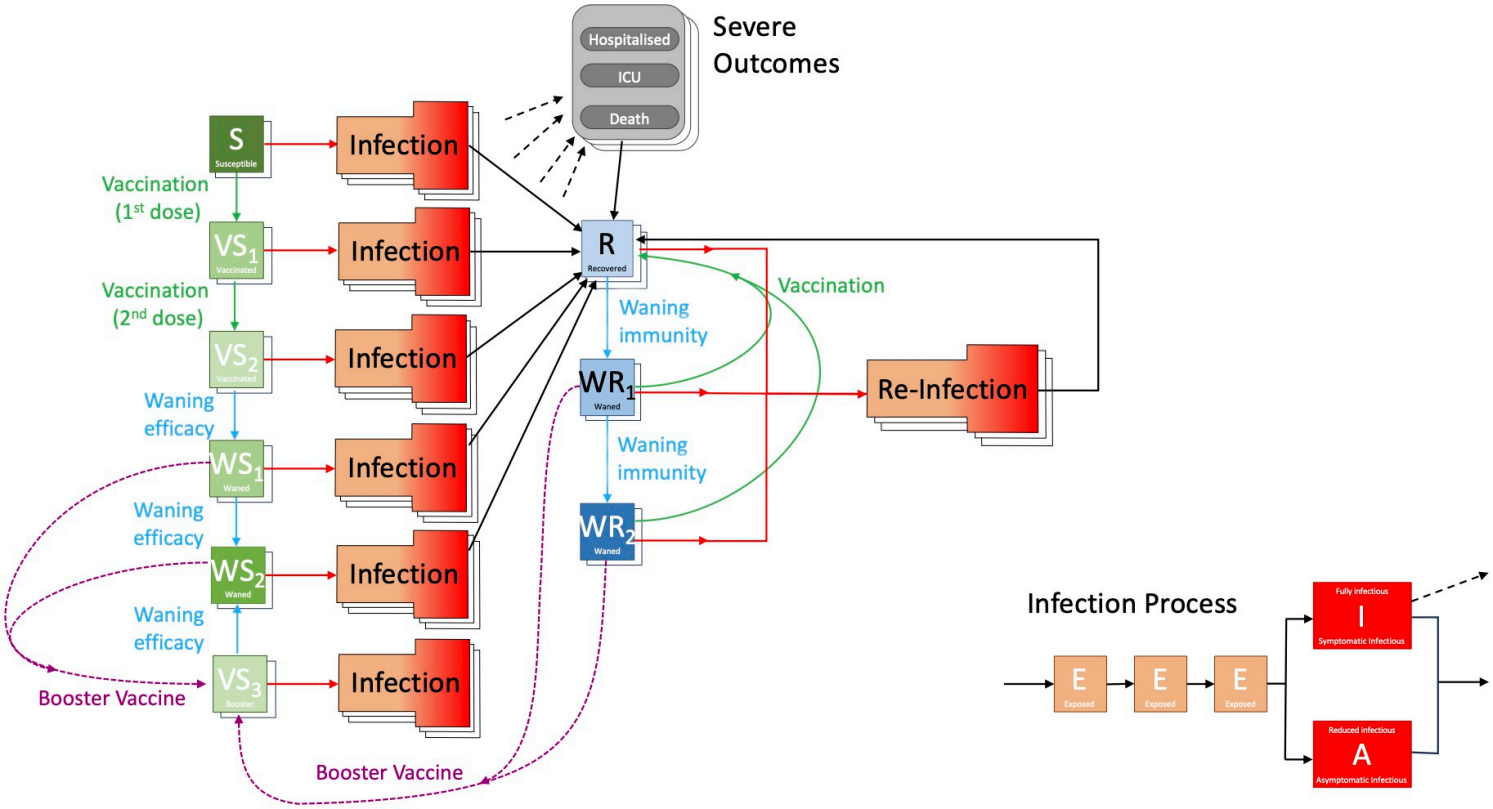
Caricatures of the development of the Warwick SARS-CoV-2 transmission and COVID-19 disease model. Red arrows show transitions due to infection, with the four compartments of the infection process shown in the bottom left. Green arrows show transitions due to vaccination, while purple dashed arrows show the transitions due to booster vaccines. The transitions due to waning immunity are shows in pale blue. Finally, recovery is shown as black arrows. Each of these states is multi-dimensional, capturing the age of individuals (single background copy of the state) or both age of the individual and infecting variant (double background copy). Severe outcomes including hospital admission, the need for ICU treatment or death are shown in a separate compartment as they do not impact on the infection dynamics.

The model is built around the traditional deterministic SEIR (Susceptible, Exposed, Infectious, Recovered) model framework [52], with three exposed classes to capture the distribution of times from infection to becoming infectious [53], and splitting the infectious group into symptomatic and asymptomatic infection (Fig 8). To this simple model we add additional structure to capture the effects of restricted social interaction during isolation whilst maintaining household transmission [33]. This fundamental model is then ‘replicated’ twenty-one times to mimic five-year age-groups (0–4, 5–9, …, 100+). The model is written as a large number of ODEs (ordinary differential equations).

This basic model was sufficient for the early waves of infection (from January to November 2020) with a single variant and without vaccination. During this early phase of the pandemic, the main driving parameter was the level of precautionary behaviour, which determined the level of social-mixing and therefore the scale of transmission outside the household [26], although we also fitted a number of other parameters (including case:hospitalisation and case:mortality ratios, age-dependent susceptibility and probability of symptoms, and the relative strength of asymptomatic compared to symptomatic transmission). From the age-structured symptomatic class, we can calculate the number of severe outcomes (hospital admissions, intensive care unit admissions and deaths), which are both key public health observables and measures of concern for this pandemic, although these quantities do not impact the transmission dynamics (Fig 8). The fitting is performed in a Bayesian framework, matching the data on the daily hospital admissions, hospital occupancy, ICU occupancy, deaths and proportion of community (Pillar 2) tests that are positive in each of the seven National Health Service (NHS) regions of England to a Poisson distribution with a mean given by the ODE model.

From late 2020, variants and vaccination increased the dimensionality of this model. Each new variant required a duplicate of all the infected model classes, to capture differences in transmission and risks of severe outcomes. The rise of each variant was captured by additionally fitting to the proportion of S-gene target failures (a proxy measure of variant-type) from TaqPath PCR testing [23, 54]. The models used in this work capture wildetype, Alpha, Delta and Omicron variants, as the main variants that affected the UK; but only the interplay between Delta and Omicron is important for the estimation although the previous variants set the level of population immunity.

The start of the vaccination campaign in December 2020 necessitated a further partitioning of the population by vaccination status (first three elements *S*, *VS*_1_ and *VS*_2_ in Fig 8 corresponding to zero, one or two doses), allowing us to capture both the reduced risk of infection and the reduced risk of severe outcomes. However, by late 2021 when Omicron invaded the UK, waning levels of protection both in terms of vaccine-induced and infection-induced immunity were added (generating additional elements in Fig 8, with waning immunity processes shown as blue arrows) and booster vaccination (shown as purple arrows in Fig 8).

We now provide a more mathematical description of the model structure and parameter inference.

### Vaccination and waning

In the absence of infection, there is still a complex pattern of vaccination and waning which is described here with the process of infection described below. The model replicates the action of:

- first and second doses of vaccine, at time-varying rates *v*_1_ and *v*_2_ respectively, that move susceptible individuals through to vaccinated states (*VS*_1_ and *VS*_2_) but have no impact on infected or recovered individuals—for model simplicity *v*_1_ and *v*_2_ correspond to the rate at which the impact of the vaccine dose takes effect, which is around 10 days post vaccination;
- waning vaccine efficacy at rates *ω*_1_ and *ω*_2_, giving a two-step process from fully vaccinated to waned efficacy—we also allow waning from state *VS*_1_ at rate $\bar{\omega }$ (where ${\bar{\omega }}^{-1}=\omega_{1}^{-1}+\omega_{2}^{-1}$), although for those that receive two doses within 3 and 12 weeks this is uncommon;
- waning immunity from past infection at rates Ω_1_ and Ω_2_ which are assumed to be slower than the waning of vaccine efficacy.

The model also needs to capture the total number of individuals who have been given a first or second dose of vaccine (*V*_1_ or *V*_2_ out of a total population size of *N*) to ensure that only individuals that have not been vaccinated are offered a first dose, and only individuals that have been vaccinated once are offered a second dose.

**Fig 9:**
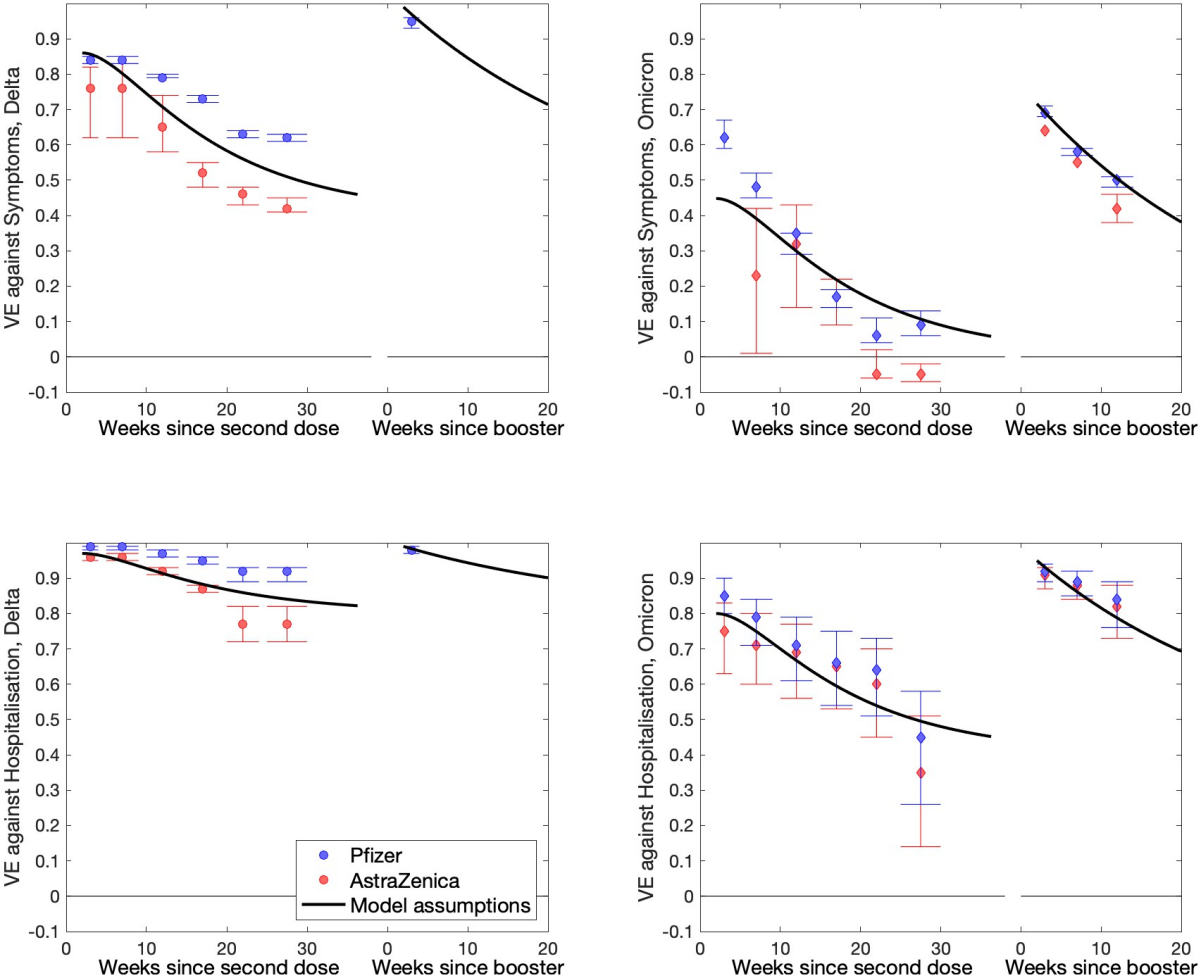
Comparison of data and model assumptions for the waning of vaccine efficacy against symptomatic infection (top) and hospital admission (bottom). Data comes from UKHSA estimates of vaccine efficacy made during late 2021 and early 2022 [29, 43], and shows waning after the second dose and waning after the booster. We separate the results by vaccine type (Pfizer in blue, AstraZenica in red) and by variant (circles for Delta in the left-hand graphs, diamonds for Omicron in the right-hand graphs). Model assumptions are presented for a 50:50 mix of Pfizer and AstraZenica vaccines in the population.

Adding subscripts that signify age (*a*) and region (*r*), when concentrating on vaccination and waning immunity (i.e. ignoring infection and variants) the underlying equations become:

$$\begin{array}{ccc} \frac{dS_{a,r}}{dt} & = & -v_{1,a,r}\frac{S_{a,r}}{N_{a,r}-V_{1,a,r}}\\ \frac{dV\;S_{1,a,r}}{dt} & = & v_{1,a,r}\frac{S_{a,r}}{N_{a,r}-V_{1,a,r}}-v_{2,a,r}\frac{V\;S_{1,a,r}}{V_{1,a,r}-V_{2,a,r}}-\bar{\omega }V\;S_{1,a,r}\\ \frac{dV\;S_{2,a,r}}{dt} & = & v_{2,a,r}\frac{V\;S_{1,a,r}}{V_{1,a,r}-V_{2,a,r}}-\omega_{1}V\;S_{2,a,r}-\omega_{B}\frac{V\;S_{2,a,r}}{V_{2,a,r}-V_{3,a,r}}\\ \frac{dW\;S_{1,a,r}}{dt} & = & \omega_{1}V\;S_{2,a,r}-\omega_{2}W\;S_{1,a,r}-\omega_{B}\frac{W\;S_{1,a,r}}{V_{2,a,r}-V_{3,a,r}}\\ \frac{dW\;S_{2,a,r}}{dt} & = & \omega_{2}W\;S_{1,a,r}+\bar{\omega }V\;S_{1,a,r}-\omega_{B}\frac{W\;S_{2,a,r}}{V_{2,a,r}-V_{3,a,r}}\\ \frac{dR_{a,r}}{dt} & = & -\Omega_{1}R_{a,r}+v_{1,a,r}\frac{W\;R_{1,a,r}+W\;R_{2,a,r}}{N_{a,r}-V_{1,a,r}}\\ \frac{dW\;R_{1,a,r}}{dt} & = & \Omega_{1}R_{a,r}-\Omega_{2}WR_{1,a,r}-v_{1,a,r}\frac{W\;R_{1,a,r}}{N_{a,r}-V_{1,a,r}}-\omega_{B}\frac{W\;R_{1,a,r}}{V_{2,a,r}-V_{3,a,r}}\\ \frac{dW\;R_{2,a,r}}{dt} & = & \Omega_{2}WR_{1,a,r}-v_{1,a,r}\frac{W\;R_{2,a,r}}{N_{a,r}-V_{1,a,r}}-\omega_{B}\frac{W\;R_{2,a,r}}{V_{2,a,r}-V_{3,a,r}}\\ \frac{dB_{a,r}}{dt} & = & v_{B,a,r}\frac{V\;S_{2,a,r}+W\;S_{1,a,r}+W\;S_{2,a,r}+W\;R_{1,a,r}+W\;R_{2,a,r}}{V_{2,a,r}-V_{3,a,r}}-\omega_{B}B_{a,r}\\ V_{1,a,r}\left(t\right) & = & \int_{0}^{t}v_{1,a,r}dt\;V_{2,a,r}\left(t\right)\;=\;\int_{0}^{t}v_{2,a,r}dt\;V_{3,a,r}\left(t\right)\;=\;\int_{0}^{t}v_{B,a,r}dt \end{array}$$ (7)

where *N*_*a*,*r*_ is the size of the population in age-group *a* and region *r*. The parameters governing vaccine waning *ω*_1_ = 100^−1^ per day, *ω*_2_ = 30^−1^ per day, and *ω*_*B*_ = 200^−1^ per day are chosen to match the estimated decline in efficacy (Fig 9). We use the same waning rates for both Omicron and Delta infections, but the initial and final levels of protection differ. We assume that waning following recovery is slow Ω_1_ = 360^−1^ per day and Ω_2_ = 1500^−1^, capturing the lack of reinfection during the early stages of the pandemic [55]; although previous infections only confer partial immunity against the Omicron variant.

### Infection dynamics

Within this section we detail the infection dynamics, including multiple exposed classes to generate an appropriate distribution for the generation time, the status of individuals with respect to their household, as well as variant and age-structure.

One of the key characteristics of the COVID-19 pandemic in the UK has been the use of self-isolation and household quarantining to reduce transmission. We approximate this process by distinguishing between first infections (caused by infection related to any non-household mixing) and subsequent household infections (caused by infection due to household mixing). We note that first infection really applies to any new infection brought into an infection free household. The first symptomatic case within a household has a probability (*H*_*t*_) of leading to household quarantining at time *t*; this curtails the non-household mixing of the individual and all subsequent infections generated by this individual. We use superscripts to denote the status of an infection with respect to this household structure: superscript *F* refers to the first infection in a household that has not been quarantined; *SI* and *SA* refer to subsequent infections that are generated by a first infection that is symptomatic or asymptomatic respectively, again in a household that has not been quarantined; *QF* refers to the first detected case in the household that leads to quarantining and *QS* is all of their subsequent household infections.

We then use subscripts to denote the multiple stages withing the exposed class, the age-group *a* of the infected individual, the region *r* and the variant *θ*:

$$\begin{array}{ccc} \frac{dE_{1,a,r,\theta }^{F}}{dt} & = & \lambda_{a,r,\theta }^{F}S_{a,r,\theta }-3\alpha_{r,\theta }E_{1,a,r,\theta }^{F}\\ \frac{dE_{1,a,r,\theta }^{SI}}{dt} & = & \lambda_{a,r,\theta }^{SI}S_{a,r,\theta }-3\alpha_{r,\theta }E_{1,a,r,\theta }^{SI}\\ \frac{dE_{1,a,r,\theta }^{SA}}{dt} & = & \lambda_{a,r,\theta }^{SA}S_{a,r},\theta -3\alpha_{r,\theta }E_{1,a,r,\theta }^{SA}\\ \frac{dE_{1,a,r,\theta }^{QS}}{dt} & = & \lambda_{a,r,\theta }^{Q}S_{a,r,\theta }-3\alpha E_{1,a,r,\theta }^{QS}\\ \frac{dE_{2,a,r,\theta }^{X}}{dt} & = & 3\alpha_{r,\theta }E_{1,a,r,\theta }^{X}-3\alpha_{r,\theta }E_{2,a,r,\theta }^{X}\\ \frac{dE_{3,a,r,\theta }^{X}}{dt} & = & 3\alpha_{r,\theta }E_{2,a,r,\theta }^{X}-3\alpha_{r,\theta }E_{3,a,r,\theta }^{X}\\ \frac{dI_{a,r,\theta }^{F}}{dt} & = & 3{\bar{d}}_{a}\left(1-H_{t}\right)\alpha_{r,\theta }E_{3,a,r,\theta }^{F}-\gamma_{\theta }I_{a,r,\theta }^{F}\\ \frac{dI_{a,r,\theta }^{SI}}{dt} & = & 3{\bar{d}}_{a}\alpha_{r,\theta }E_{3,a,r,\theta }^{SI}-\gamma_{\theta }I_{a,r,\theta }^{SI}\\ \frac{dI_{a,r,\theta }^{SA}}{dt} & = & 3{\bar{d}}_{a}\left(1-H_{t}\right)\alpha_{r,\theta }E_{3,a,r,\theta }^{SA}-\gamma_{\theta }I_{a,r,\theta }^{SA}\\ \frac{dI_{a,r,\theta }^{QF}}{dt} & = & 3{\bar{d}}_{a}H_{t}\alpha_{r,\theta }E_{3,a,r,\theta }^{F}-\gamma_{\theta }I_{a,r,\theta }^{QF}\\ \frac{dI_{a,r,\theta }^{QS}}{dt} & = & 3{\bar{d}}_{a}\alpha_{r,\theta }E_{3,a,r,\theta }^{QS}+3d_{a,\theta }H_{t}\alpha_{r,\theta }E_{3,a,r,\theta }^{SA}-\gamma_{\theta }I_{a,r,\theta }^{QS}\\ \frac{dA_{a,r,\theta }^{X}}{dt} & = & 3\left(1-{\bar{d}}_{a}\right)\alpha_{r,\theta }E_{3,a,r,\theta }^{X}-\gamma_{\theta }A_{a,r,\theta }^{X}\\ \frac{dR_{a,r,\theta }}{dt} & = & \gamma_{\theta }\sum_{X,\theta }\left(I_{a,r,\theta }^{X}+A_{a,r,\theta }^{X}\right) \end{array}$$ (8)

where

X∈{F,SI,SA,QF,QS}

where

$$\begin{array}{ccc} \lambda_{a,r,\theta }^{F} & = & \sigma_{a}{\hat{\beta }}_{\theta }\sum_{b}(\beta_{b,a,t}^{S}+\beta_{b,a,t}^{W}+\beta_{b,a,t}^{O})(I_{b,r,\theta }^{F}+I_{b,r,\theta }^{SI}+I_{b,r,\theta }^{SA}+\tau A_{b,r,\theta }^{F}\\ & & \;+\tau A_{b,r,\theta }^{SI}+\tau A_{b,r,\theta }^{SA})\\ \lambda_{a,r,\theta }^{SI} & = & \sigma_{a}{\hat{\beta }}_{\theta }\sum_{b}\beta_{b,a,t}^{H}I_{b,r,\theta }^{F},\;\\ \lambda_{a,r,\theta }^{SA} & = & \sigma_{a}{\hat{\beta }}_{\theta }\sum_{b}\beta_{b,a,t}^{H}\tau A_{b,r,\theta }^{F},\;\\ \lambda_{a,r,\theta }^{Q} & = & \sigma_{a}{\hat{\beta }}_{\theta }\sum_{b}\beta_{b,a,t}^{H}I_{b,r,\theta }^{QF} \end{array}$$

Here S is a measure of the susceptible population (including both naive, vaccinated, waned, boosted and recovered individuals, scaled by their relative susceptibility against the variant); λ refers to the force of infection generating first infections (superscript *F*), secondary infections within the home from a symptomatic or asymptomatic first infection (superscript *SI* or *SA*) or from quarantined individuals (superscript *Q*); *α* is the rate of movement from exposed to infectious and *γ* is the recovery rate; *τ* is the reduced level of transmission from asymptomatic infection relative to symptomatic infection; $\bar{d}$ is the probability of that an infection will be symptomatic (which is dependent on the vaccine status of infected individuals), and *H*_*t*_ is the probably that a symptomatic infection will lead to household quarantining. The dependence of these parameters on age (*a*), region (*r*) and variant (*θ*) is explicitly shown; only *H*_*t*_ is time-dependent and is assumed to be related to the level of precautionary behaviour *ϕ*_*t*_.

The force of infection, λ, is again partitioned by whether the individual getting infected is the first, subsequent or from a quarantined household. This risk of infection is driven by the age-dependent mixing matrices for home, school, work and other contacts (***β***^*H*^, ***β***^*S*^, ***β***^*W*^ and ***β***^*O*^ respectively) which scale with the estimated time-dependent precautionary behaviour. The risk of infection also varies with the variant (as captured by ${\hat{\beta }}_{\theta }$) and the age-dependent risk of infection (*σ*_*a*_).

### Linking infection, vaccination dynamics and outcomes

To link together the previous two model sections, we need to focus on the status of those individuals who get infected. We define a vector of potentially susceptible groups:

$$\Psi_{a,r}=(S_{a,r},V\;S_{1,a,r},V\;S_{2,a,r},W\;S_{1,a,r},W\;S_{2,a,r},B_{a,r},R_{a,r,theta},W\;R_{1,a,r},W\;R_{2,a,r})$$

The total susceptible variable that feeds into the infection Eq (8) is then the dot product of this susceptible vector with the vector of susceptibility to infection:

$$\begin{array}{c} S_{a,r,\theta }=\Psi_{a,r}\cdot \sigma_{\theta }^{I}\;\;\text{where}\;\;\sigma_{\theta }^{I}=(\sigma_{S,\theta },\sigma_{V1,\theta }^{I},\sigma_{V2,\theta }^{I},\sigma_{V2,\theta }^{I},\sigma_{W,\theta }^{I},\sigma_{R,\theta }^{I},\sigma_{R,\theta }^{I},\sigma_{R2,\theta }^{I}) \end{array}$$

noting that the susceptibility of those in the first waned compartment (*WS*_1_) is the same as for those who have received both doses of vaccine (*VS*_2_), and that the risk of infection to those in the recovered or first waned compartment after recovery is zero for all pre-Omicron variants. The values of the susceptibility *σ*^*I*^ is one minus the protection against infection, with the weighting between the protection afforded by AstraZeneca and by mRNA vaccines (Pfizer or Moderna) given by the age and region specific ratio of the vaccines delivered up to that point in time.

Similarly, the risk of symptoms (${\bar{d}}_{a}$) used in Eq (8) is also based on the vector of susceptiblities:

$$\begin{array}{c} {\bar{d}}_{a}=D_{a}\;\Psi_{a,r}\cdot \sigma_{\theta }^{D}\;\;\text{where}\;\;\sigma_{\theta }^{D}=(\sigma_{S,\theta },\sigma_{V1,\theta }^{D},\sigma_{V2,\theta }^{D},\sigma_{V2,\theta }^{D},\sigma_{W,\theta }^{D},\sigma_{R,\theta }^{D},\sigma_{R,\theta }^{D},\sigma_{R2,\theta }^{D}) \end{array}$$

In this case *σ*^*d*^ is the ratio of one minus the protection against symptoms relative to the one minus the protection against infection, and *D*_*a*_ is the age-dependent risk of developing symptoms (extracted from the early case-reporting data [33]).

A similar approach is used to determine the number of hospital admission, the number of ICU admissions, the number of deaths and the level of hospital and ICU occupancy. We first define the rate of generating newly symptomatic infectious individuals as:

$$N\;I_{a,r,\theta }\left(t\right)=3{\bar{d}}_{a}\alpha_{r,\theta }\sum_{X}E_{3,a,r,\theta }^{X}\left(t\right)$$

The modelled rate of admission to hospital or admission to ICU is then given by:

$$\begin{array}{ccc} M_{a,r,\theta }^{HospAd}\left(t\right) & = & \int_{0}^{\infty }H_{a}{\hat{H}}_{r,\theta }T^{H}\left(\tau \right)N\;I_{a,r,\theta }\left(t-\tau \right)d\tau \\ M_{a,r,\theta }^{ICUAd}\left(t\right) & = & \int_{0}^{\infty }I_{a}{\hat{I}}_{r,\theta }T^{I}\left(\tau \right)N\;I_{a,r,\theta }\left(t-\tau \right)d\tau \end{array}$$

where each term consists of an age-dependent risk (*H*_*a*_ and *I*_*a*_, taken from the early data), a regional and variant dependent scaling factor (${\hat{H}}_{r,\theta }$ and ${\hat{I}}_{r,\theta }$, estimated through our fitting procedures) and a delay between the onset of symptoms and the epidemiological event (*T*^*H*^(*τ*) and *T*^*I*^(*τ*), with these distributions based on recorded data). The modelled death rate follows a similar form, but is amplified by high levels of hospital occupancy in a region relative to the population size ($M_{r}^{HospOcc}/N_{r}$):

$$M_{a,r,\theta }^{Death}\left(t\right)=(1+F_{r}\frac{M_{r}^{HospOcc}}{N_{r}})\int_{0}^{\infty }D_{a}{\hat{D}}_{r,\theta }T^{D}\left(\tau \right)N\;I_{a,r,\theta }\left(t-\tau \right)d\tau$$

where *F*_*r*_ is estimated at 1600 (CI 770-2800), such that the death rate approximately doubles if 0.06% of a region is in hospital—which is close to the levels observed at the peak of the Alpha wave.

Hospital and ICU occupancy are then computed based on the recorded distributions (*D*) of length of stays [33]:

$$\begin{array}{ccc} M_{a,r,\theta }^{HospOcc}\left(t\right) & = & \int_{0}^{\infty }D_{a}^{H}\left(\tau \kappa_{r,\theta }^{H}\right)M_{a,r,\theta }^{HospAd}\left(t-\tau \right)d\tau \\ M_{a,r,\theta }^{ICUOcc}\left(t\right) & = & \int_{0}^{\infty }D_{a}^{I}\left(\tau \kappa_{r,\theta }^{I}\right)M_{a,r,\theta }^{ICUAd}\left(t-\tau \right)d\tau \end{array}$$

These distributions are scaled for each age-group and variant (by a factor *κ*); We note that children and young adults spending less average time in hospital and the average length of stay being longer for Alpha and Delta variants than for the wildtype variant.

### Precautionary behaviour

One of the main parameters that drives much of the dynamics is the level of precautionary behaviour *ϕ*_*t*,*r*_. We view *ϕ*_*t*,*r*_ as slowly varying (except when there is an abrupt change in policy) and captures how the risky interactions between susceptible and infectious individuals scale throughout the pandemic. As such the precautionary behaviour captures the changes in social mixing (including working from home) as well as behavioural changes such as mask-use and isolation. The level of precautionary behaviour is estimated for each week and each region as part of our fitting procedure (see below) and is a scalar parameter between zero and one; when *ϕ* = 0 we have returned to pre-pandemic mixing whereas *ϕ* = 1 corresponds to a stringent lock-down. In particular, *ϕ*_*t*_ is used to regulate the home, work, school and other transmission matrices:

$$\begin{array}{cc} \beta_{b,a,t}^{H} & ={\tilde{\beta }}_{a,b}^{H}[\left(1-\phi_{t}\right)+\phi_{t}q^{H}]\\ \beta_{b,a,t}^{S} & ={\tilde{\beta }}_{a,b}^{S}[\left(1-\phi_{t}\right)+\phi_{t}q^{S}]\\ \beta_{b,a,t}^{W} & =\left(1-f\right){\tilde{\beta }}_{a,b}^{W}[\left(1-\phi_{t}\right)+\phi_{t}q^{W}]+f{\tilde{\beta }}_{a,b}^{W}(\left(1-\phi_{t}\right)+\phi_{t}q^{W})\left(\left(1-\phi_{t}\right)+\phi_{t}q^{O}\right)\\ \beta_{ba}^{O} & ={\tilde{\beta }}_{b,a}^{O}{\left(\left(1-\phi_{t}\right)+\phi_{t}q^{O}\right)}^{2} \end{array}$$

Here, $\tilde{\beta }$ are the mixing matrices for home, school, work and other contacts during pre-pandemic circumstances as given by [56]; and *q* acts to define the scaling during severe retardation of social mixing (*q*^*H*^ = 1.25 such that within household mixing increases during lockdown, *q*^*W*^ = 0.2 such that some work activities have to continue, *q*^*S*^ = 0.05 and *q*^*O*^ = 0.05). For work contacts we separate industries that are public-facing (*f* = 0.3, such a leisure and retail) from other employment; contacts in public-facing industries are assumed to scale with quadratically accounting for both the number of individuals at work and the number of people accessing these activities.

**Fig 10:**
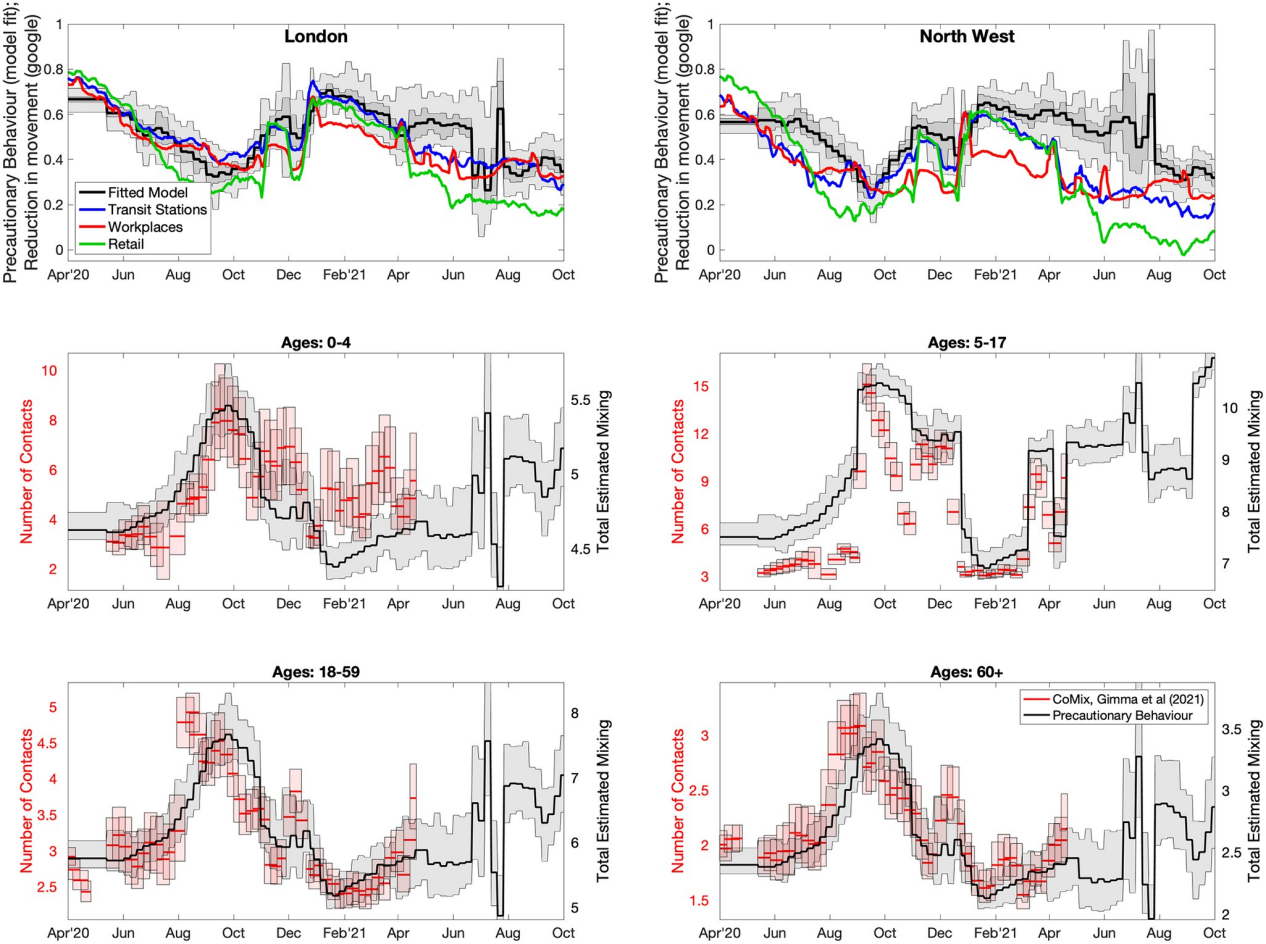
Comparison between estimated measures of social mixing with the model (black) and recorded observations from google mobility [57] and CoMix [58]. In the top two panels we directly compare the level of precautionary behaviour *ϕ*_*t*,*r*_ with the reduction in movements (transit stations in blue, workplaces in red and retail in green) as estimated from google mobility [57] observations. This is shown for London and the North West; there is better qualitative agreement for London which may reflect the degree to which recorded movements capture population-level mixing in the capital. In the lower four panels we qualitatively compare the number of contacts recorded by CoMix [58] in four age-groups (0-4, 5-17, 18-59 and 60+) with the estimated mixing from our age-structured transmission matrices. Given the mixing matrices are re-scaled to generated a transmission rate we would not expect a one-to-one agreement with the recorded number of contacts.

While the level of precautionary behaviour is inferred by matching to epidemiological data, it is interesting to compare the estimates to other more directly recorded measures of behaviour. In Fig 10 we compare our estimated values to google-mobility data [57] (top two panels) and diary-based records of contacts [58] (lower four panels). For the google mobility comparison, we consider the estimated precautionary behaviour (*ϕ*_*t*_) in comparison to the reduction in movements as measures by google over this period. We focus on two regions, London and the North West, noting that while both have similar features, the qualitative agreement for London is far stronger. When comparing to the Co-Mix study [58], the available data is number of contacts per person in a given age-group, we therefore compare this to the mixing matrices for those ages (e.g. $\sum_{b}\beta_{b,a,t}^{H}+\beta_{b,a,t}^{S}+\beta_{b,a,t}^{W}+\beta_{b,a,t}^{O}$ for age group *a*), noting that as these measure different things there will not be a one-to-one matching. We again find that our estimated mixing agrees with the age-structured trends identified by Co-Mix. A more thorough description of the Precautionary Behaviour and the different behavioural elements is given in [27].

### Parameter inference

Key to the accuracy of any model are the parameters that underpin the dynamics. With a model of this complexity, a large number of parameters are required. Some, such as vaccine efficacy and waning, are taken from current literature; while others are inferred from the epidemic dynamics.

Of the inferred parameters there are three basic classes; those, such as scalings of the case-hospitalisation ratios, that are different between regions and variants; others such as age-dependent susceptiblity are universal (the same for all regions and variants); while the level of precautionary behaviour over time changes on a weekly time-scale. Bayesian inference, using an MCMC process, is applied to each of the seven NHS regions in England to determine posterior distributions for each of the regional parameters (further details are given in [26]). The distribution of parameters leads to uncertainty in model projections, which is represented by the 95% prediction interval in all graphs (this interval contains 95% of all predictions). We note that when we compare two scenarios (for example vaccination with a 3-week interval, with vaccination using a 12-week interval) we compared simulations with the same parameters chosen from the posterior distributions—and then calculated means and 95% prediction intervals based on these results.

As the epidemic has progressed, new posterior distributions based on the latest data are initialised from previous MCMC chains—ensuring a rapid fit to historical data. In general this refitting process has been performed weekly (or twice weekly) throughout the pandemic. For the time period of relevance in this paper (December 2020—September 2021), we matched to seven observations: hospital admissions, hospital occupancy, ICU occupancy (noting that data on ICU admissions is not available), deaths, proportion of pillar 2 (community) test that are positive, the proportion of pillar 2 tests that are S-gene negative (as a signal of the ratio of wild-type to Alpha variant, then a signal of the ratio of Delta to Alpha variant), and the early REACT data as a measure of sero-prevalence [59]. As such our log-likelihood function is given by:

$$\begin{array}{c} \begin{array}{c} \text{LogLike}({\text{Data}}_{r}|\Theta_{r})\;=\;\\ \;\sum_{t}l\;\;\;P\left(\text{Hospital}\;{\text{Admissions}}_{r}\left(t\right)|\sum_{a,\theta }M_{a,r,\theta }^{HospAd}\left(t\right)\right)\;+\\ \;l\;\;\;P({\text{Deaths}}_{r}\left(t\right)|\sum_{a,\theta }M_{a,r,\theta }^{Death}\left(t\right))\;+\\ \;l\;\;\;P(\text{Hospital}\;{\text{Occupancy}}_{r}\left(t\right)|\sum_{a,\theta }M_{a,r,\theta }^{HospOcc}\left(t\right))\;+\\ \;l\;\;\;P(\text{ICU}\;{\text{Occupancy}}_{r}\left(t\right)|\sum_{a,\theta }M_{a,r,\theta }^{ICUOcc}\left(t\right))\;+\\ \;l\;\;\;B(\text{Positive}\;{\text{Tests}}_{r}\left(t\right)|\text{Total}\;{\text{Tests}}_{r}\left(t\right),\sum_{a,\theta }F_{\theta }N\;I_{a,r,\theta }\left(t\right)/\sum_{a}N_{a,r})\;+\\ \;l\;\;\;B(\text{S-gene}\;\text{Neg}\;{\text{Tests}}_{r}\left(t\right)|\text{Total}\;{\text{Tests}}_{r}\left(t\right),\sum_{a}N\;I_{a,r,\text{Alpha}}\left(t\right)/\sum_{a,\theta }N\;I_{a,r,\theta }\left(t\right))\;+\\ \;l\;\;\;B(\text{REACT}\;{\text{Sero}}_{r}\left(t\right)|\text{REACT}\;{\text{Tests}}_{r}\left(t\right),\sum_{a,\theta }\int_{0}^{t}N\;I_{a,r,\theta }\left(\tau \right)d\tau /\sum_{a}N_{a,r}) \end{array} \end{array}$$ (9)

where *lP* and *lB* are the logs of Poisson and Binomial probabilities, *N*_*a*,*r*_ is the population size of individuals in age-group *a* and region *r*, *F*_*θ*_ is a variant dependent level of reporting, and **Θ**_*r*_ is the set of all model parameters for region *r*. We note that in [26], which was written in the early stages of the pandemic, we did not fit to S-gene data as we had been dealing with a single variant. Although not part of the underlying transmission dynamics, the seven quantities for each spatial region can be generated from the number, age and type of infection within the model, as described above.

**Fig 11:**
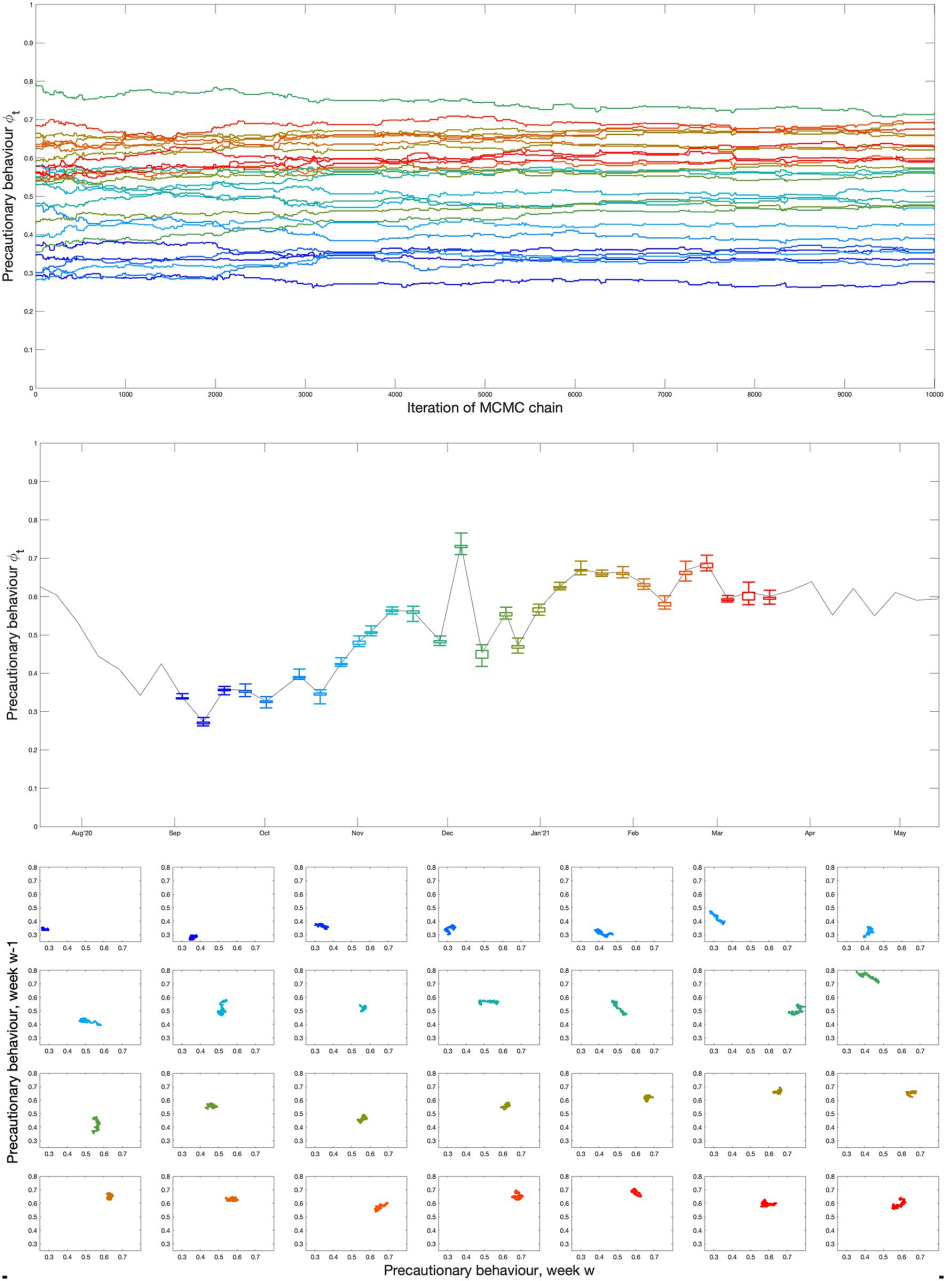
Example of an MCMC chain for *ϕ*_*t*_ in the Midlands from the main Alpha wave. Throughout, the plotted data is colour-coded (from blue to red) corresponding to the date it represents. The top panel show the behaviour of the 29 weekly values of *ϕ* across 10,000 iterations of the chain; the central panel shows the mean *ϕ* values (line) together with the inter-quartile and 95% credible interval for each week, again taken from 10,000 iterations of the chain; while the lower panel shows the correlation between the value of *ϕ* against the value the previous week.

Fig 11 shows an example of one chain for the precautionary behaviour, *ϕ*_*t*,*r*_, during the period September 2020 to March 2021 that captures the bulk of the Alpha wave. The chain is 10,000 iterations of the MCMC process, and refers to the precautionary behaviour for the Midlands region; a typical chain from the weekly re-fitting is around 3-5,000 iterations, whereas the chains used are all 20,000 iterations or longer. The figure shows the individual chains for the 29 weekly values of *ϕ*_*t*_ (top panel); the mean, inter-quartile range and 95% credible intervals for each parameter through time; and the correlation between *ϕ*_*t*_ and the value the preceding week.

When fitting to the BA.1 Omicron outbreak, we have already seen in the main paper that our prior assumptions can be key—especially in the early stages of an outbreak when there is insufficient data to inform the posterior. In Table 3 we provide the priors used in the inference which are specified as a scaling on the parameters already inferred for the Delta variant.

**Table 3:** Prior distributions assumed for the full Omicron model; these prior distributions are shown in Fig 7 as shaded regions.

Parameter	Prior
$R_{0}^{\text{Omicron}}$	$R_{0}^{\text{Delta}}\left(1+\text{Exp}\left(1/3\right)\right)$
Periods^Omicron^	$1.2\times {\text{Periods}}^{\text{Delta}}\beta \left(1.5,1.1\right)$
Severity^Omicron^	$1.2\times {\text{Severity}}^{\text{Delta}}\beta \left(1.5,1.1\right)$
Immune Escape, *Q*^Omicron^	(1 − *Q*)^2^, *Q* ∈ [0, 1]
